# High-throughput behavioral phenotyping in the expanded panel of BXD recombinant inbred strains

**DOI:** 10.1111/j.1601-183X.2009.00540.x

**Published:** 2010-03

**Authors:** V M Philip, S Duvvuru, B Gomero, T A Ansah, C D Blaha, M N Cook, K M Hamre, W R Lariviere, D B Matthews, G Mittleman, D Goldowitz, E J Chesler

**Affiliations:** † Systems Genetics Group, Biosciences Division, Oak Ridge National LaboratoryOak Ridge TN; ‡ Department of Neurobiology and Neurotoxicology, Meharry Medical CollegeNashville, TN; § Department of Psychology, The University of MemphisMemphis, TN; ¶ Departments of Anatomy and Neurobiology, University of Tennessee Health Science CenterMemphis, TN; ** Departments of Anesthesiology and Neurobiology, University of Pittsburgh School of MedicinePittsburgh, PA; †† Departments of Psychology and Neuroscience, Baylor UniversityWaco, TX, USA; ‡‡ Present address: Department of Psychology, Nanyang Technological UniversitySingapore; §§ Centre for Molecular Medicine and Therapeutics, Department of Medical Genetics, University of British ColumbiaVancouver, BC, Canada

**Keywords:** gene expression, heritability, sex differences, systems genetics

## Abstract

Genetic reference populations, particularly the BXD recombinant inbred (BXD RI) strains derived from C57BL/6J and DBA/2J mice, are a valuable resource for the discovery of the bio-molecular substrates and genetic drivers responsible for trait variation and covariation. This approach can be profitably applied in the analysis of susceptibility and mechanisms of drug and alcohol use disorders for which many predisposing behaviors may predict the occurrence and manifestation of increased preference for these substances. Many of these traits are modeled by common mouse behavioral assays, facilitating the detection of patterns and sources of genetic coregulation of predisposing phenotypes and substance consumption. Members of the Tennessee Mouse Genome Consortium (TMGC) have obtained phenotype data from over 250 measures related to multiple behavioral assays across several batteries: response to, and withdrawal from cocaine, 3,4-methylenedioxymethamphetamine; “ecstasy” (MDMA), morphine and alcohol; novelty seeking; behavioral despair and related neurological phenomena; pain sensitivity; stress sensitivity; anxiety; hyperactivity and sleep/wake cycles. All traits have been measured in both sexes in approximately 70 strains of the recently expanded panel of BXD RI strains. Sex differences and heritability estimates were obtained for each trait, and a comparison of early (*N* = 32) and recent (*N* = 37) BXD RI lines was performed. Primary data are publicly available for heritability, sex difference and genetic analyses using the MouseTrack database, and are also available in GeneNetwork.org for quantitative trait locus (QTL) detection and genetic analysis of gene expression. Together with the results of related studies, these data form a public resource for integrative systems genetic analysis of neurobehavioral traits.

BXD recombinant inbred (BXD RI) mice are an established behavior genetics resource, often used for the study of alcoholism and other neuropharmacological traits (Crabbe *et al.* 1996; Gora-Maslak *et al.* 1991; Plomin *et al.* 1991). These lines have been used for three decades to map the genetic basis of complex phenotypes, and allow detection of causative genetic loci even for traits with modest heritability (Belknap 1998). The population also serves as a genetic reference population, allowing correlation and comparison across traits, both within and among different laboratories to evaluate common genetic determinants of correlated phenotypes (Crabbe *et al.* 1996). This approach has been facilitated through the development of GeneNetwork (www.genenetwork.org), an Internet resource for the multi-variate genetic analysis of complex traits in genetic reference populations (Chesler *et al.* 2003, 2004; Wang *et al.* 2003). GeneNetwork aids in identification of candidate genes and bio-molecular mechanisms underlying addiction-related phenotypes and includes a wealth of data on mRNA expression profiles from various tissues of the central nervous system (Chesler *et al.* 2005; Peirce *et al.* 2006; Rosen *et al.* 2003, 2007). Despite a wealth of data from many previous studies in these lines, the potential for integrative multi-variate analysis has been limited by the depth and breadth of previous behavioral phenotyping.

The BXD RI lines were initially derived by B. A. Taylor (e.g. Taylor *et al.* 1977) through inbreeding the progeny of an intercross of C57BL/6J mice (B6) and DBA/2J mice (D2). Additional lines were added in 1999 (Taylor *et al.* 1999), resulting in a set of approximately 35 strains. A recent expansion has increased the population to 79 lines in total (Peirce *et al.* 2004). The new lines are derived from an advanced intercross implemented as described by Darvasi and Soller (1995) and have a higher number of recombinations per line, allowing an increase in the precision with which quantitative trait loci (QTL) can be detected (Shifman *et al.* 2006). Although the BXD RI lines are becoming more widely used, many common behavioral phenotypes have not been studied to date, have been measured in only a few lines or have only been studied in one sex.

The present study is one of several ongoing efforts that will allow for an integrative multi-variate analysis through the collection of a large set of broad-based behavioral phenotyping data in the newly expanded BXD RI strain population. It emphasizes behavioral predictors of susceptibility to substance use disorders. The same genetic polymorphisms and environmental interactions that influence predisposing phenotypes may also influence preference and addiction-related traits including drug self-administration and withdrawal. In this study, we have focused on potential predisposing phenotypes including stress or pain sensitivity, anxiety, despair, hyperactivity and abnormal circadian rhythms, pharmacokinetic and pharmacodynamic responses to drugs of abuse, including withdrawal, sensitization, activity effects, anxiolytic and neurological effects including neurogenesis. By making these data public, we further hope to provide a resource of neurobehavioral phenotype data in the expanded BXDs that complement existing molecular phenotype data for systems genetic analysis of brain and behavior.

## Materials and methods

### Multi-variate phenotyping batteries

In order to rapidly develop a broad base of behavioral phenotyping data, phenotyping was performed in several multi-variate test batteries (Table 1). Each battery consists of a set of tests administered serially to individual mice. This approach also allows for the examination of partial-correlation within strain (non-genetic correlation) but may upwardly bias estimates of genetic correlation obtained using strain means of the measures. A given mouse was assigned to one and only one battery and received all tests in that battery. The order of repeated testing (reported in Table 1) was either fixed where logical and necessary or, in the case of the nociception battery, varied systematically using randomly generated Latin-square designs each applied to a different strain and sex. In general, for fixed-order batteries, the least stressful measures were obtained first, and all baseline measures were necessarily obtained before conditioning or drug exposures. The testing protocols were largely derivative of those developed in consultation with the external advisory board of the Tennessee Mouse Genome Consortium (TMGC) ENU-Neuromutagenesis Program (Goldowitz *et al.* 2004).

**Table 1:** Behavioral assays within each phenotyping battery

				Sample sizes within strain		
Battery (site)	Test description	Apparatus (Mfg, model, dimensions)	Test order	Average	Max	Number of strains
Adrenals (UTHSC)	Left and right adrenal weights	Zeiss Dissecting Microscope, Mettler Toledo scale, Bouin's Fixative, Paraplast Plus Tissue Embedding Medium (McCormick Scientific, St. Louis, MO, USA)	Single measure	4♂, 4♀	8♂, 8♀	62♂, 63♀
Adult neurogenesis (UTHSC)	Number of BrdU+ cells per millimeter length in the RMS	Vectastain Elite ABC Kit (Vector Laboratories, Burlingame, CA, USA) AnalySIS Opti Version 3.3.776	Single measure	3♂, 3♀	5♂, 5♀	40♂, 36♀
Cocaine (U. Memphis)	Cocaine-conditioned place preference; locomotor activity in a novel environment, after saline, and after each of two cocaine treatments	Eight Med-Associates automated open field chambers (43.2 cm L × 43.2 cm W × 30.4 cm H; part no. ENV-515), Eight Med-Associates place preference chambers (46 cm L × 14 cm W × 20 cm H; part no. ENV-3013)	Fixed: OF-novel, OF-saline, OF-cocaine, OF-cocaine 2, cocaine place preference; morphine withdrawal (naïve set)	11♂, 10♀	21♂, 28♀	66♂, 64♀
Ethanol 1 (UTHSC)	OFA after saline and ethanol; elevated plus maze after saline and ethanol; rotarod ataxia	Columbus Instruments Rotarod and Plus Maze, AccuScan Activity Chamber, Razor blade, Analox tube, Centrifuge	Ethanol first in half of mice, saline first in half of mice, fixed: OFA, EPM, rotarod	4♂, 3♀	6♂, 6♀	56♂, 48♀
General behavior (U. Memphis)	Hot plate; light–dark box, zero-maze; open field activity; pre-pulse inhibition; acoustic startle response	AccuScan Instruments Elevated Zero-Maze, Hamilton-Kinder Smart Frame Monitor with Open Field and Light/Dark Insert, IITC Hot-Plate Unit, Hamilton-Kinder SM100 Startle Monitors	Fixed: Hot plate, light-dark box, zero-maze, open field activity, acoustic startle response, pre-pulse inhibition	10♂, 9♀	29♂, 32♀	60♂, 58♀
MDMA (Meharry)	Open field activity in a novel environment; locomotor activity after saline or MDMA	Med Associates Open Field Activity System MED-OFA-510, Digital video camera	Fixed: OF, FST	2♂, 2♀	4♂, 2♀	20♂, 15♀
Alcohol withdrawal (U. Memphis)	HICs–baseline and at 1 h intervals after EtOH	N/A	Fixed: baseline, post-injection intervals	5♂, 5♀	14♂, 12♀	36♂, 30♀
Morphine (U of Memphis)	Open field activity in a novel environment and after morphine, salivation, defecation, urination, postural effects, wet dog shakes and ptosis after morphine treatment	Eight Med-Associates automated open field chambers (43.2 cm × 43.2 cm; part no. ENV-515)	Fixed: OF after saline, OF after morphine	6♂, 6♀	21♂, 28♀	62♂, 62♀
Pain (ORNL)	Hargreaves' test, hot plate, tail withdrawal, tail clip, von Frey test	IITC Life Science Inc., 336 TG Plantar Test (Hargreaves) and Tail Flick Analgesia Meter with Heated Glass and Tail Temperature, IITC Life Science Inc., Hot-Plate Analgesia Meter, Boekel Scientific/Grant Optima Immersion Circulator Model GR150, 600g Alligator Clamp w/Heat-Shrink coating, von Frey Fiber Set Stoelting Inc.	Unique Latin Square within each strain and sex	7♂, 6♀	10♂, 10♀	60♂, 54♀
Ethanol 2 (UTHSC)	Dowel test, Porsolt forced swim test	Piezo System, Swimming Chamber, Dowel, LX-101 Lux meter	Fixed: sleep, Porsolt, Dowel	4♂, 4♀	11♂, 7♀	55♂, 47♀
Vocalization (U of Memphis)	Footshock vocalization threshold	Med Associates Inc., Shock Titration Package for Mice (model ENV-307 W)	Single measure, repeated shock with increasing mA intensity	5♂, 5♀	9♂, 8♀	67♂, 66♀

### Subjects

A range of 3–11 mice per sex per strain from new and historical BXD RI lines were characterized for each phenotype (Table 1). Testing occurred at 8–9 weeks of age. Within each strain, mice came from at least two litters, with some, but never all, males and females from the same litter. Litter information is stored in the MouseTrack system and can be obtained for further modeling.

Approximately 70 strains were available for phenotyping, allowing improved power for QTL mapping and genetic correlation. BXD 1–42/TyJ strains were obtained from the Jackson Laboratory (Bar Harbor, ME, USA). Recent BXD RI lines (Peirce *et al.* 2004) were provided by Dr Lu Lu and Dr Robert W. Williams (University of Tennessee Health Science Center, Memphis, TN, USA).

Housing and testing environment conditions were maintained throughout testing. Except where noted, BXD RI lines were imported into the Russell Vivarium at Oak Ridge National Laboratory (ORNL) for environmentally controlled, year round breeding and distribution for all assays except for handling-induced convulsion (HIC) and footshock vocalization, for which mice were bred at University of Tennessee Health Science Center (UTHSC) and housed as described in Matthews *et al.* (2008). Litters were weaned at about 3 weeks of age and shipped to various test sites in the TMGC's climate controlled, specific pathogen-free (SPF) mouse transport van at about 6–7 weeks of age, allowing at least 1 week of acclimation to their new home colony. Animals were transported in static micro-isolators. Housing conditions, apparatus information and testing protocols specific to each battery are summarized in Table 2. All mice were housed in rooms lit with fluorescent ceiling lights and had Harlan Softcob bedding. No other species were present in the room and mice received daily health checks.

**Table 2:** Housing conditions, apparatus information and testing protocols across test sites

Battery	Breeding	Pain	MDMA	Alcohol withdrawal	Cocaine	General behavior	Morphine	Adrenals/ adult neurogenesis	Ethanol 1	Ethanol 2	Sleep	Vocalization

Site	ORNL	ORNL	Meharry	U of Memphis	U of Memphis	U of Memphis	U of Memphis	UTHSC	UTHSC	UTHSC	UTHSC	U of Memphis
Caging system												
Cage distributor, type, material, size	Optimice Single (75 sq. in.), Thoren Single (75 sq. in.), Thoren Duplex (52 sq. in.). All poly-carobnate	Optimice Single (poly-carbonate, 75 sq. in.)	Generic Static Micro-isolators 18.5 × 29.5 cm poly-carbonate	Generic Static Micro-isolators 18.5 × 29.5 cm poly-carbonate	Generic Static Micro-isolators 18.5 × 29.5 cm poly-carbonate	Generic Static Micro-isolators 18.5 × 29.5 cm poly-carbonate	Generic Static Micro-isolators 18.5 × 29.5 cm poly-carbonate	Alternative Design Static Micro-isolators 18.5 × 29.5 cm poly-carbonate	Standard mouse shoebox cages 7.25 × 11.5 in. of poly-carbonate/ polysulfone	Generic Static Micro-isolators 18.5 × 29.5 cm poly-carbonate	54 sq. in. plexiglass box made at University of Kentucky	Generic Static Micro-isolators 18.5 × 29.5 cm poly-carbonate
Lid distributor, type, material, size	Optimice poly-carbonate top, Thoren filter top	Optimice poly-carbonate top	Laboratory products poly-carbonate ‘One Cage’ Micro-isolator filter top	Generic Static Micro-isolators 18.5 × 29.5 cm poly-carbonate	Generic Static Micro-isolators 18.5 × 29.5 cm poly-carbonate	Generic Static Micro-isolators 18.5 × 29.5 cm poly-carbonate	Generic Static Micro-isolators 18.5 × 29.5 cm poly-carbonate	Alternative designs, poly-carbonate	Standard mouse cage grilles of stainless steel	Standard mouse cage grilles of stainless steel	Same as cage	Generic Static Micro-isolators 18.5 × 29.5 cm poly-carbonate
Filter top, distributor, type	Optimice poly-carbonate top, Thoren filter top	Optimice poly-carbonate top	Laboratory products poly-carbonate ‘One Cage’ Micro-isolator filter top	Generic Static Micro-isolators 18.5 × 29.5 cm poly-carbonate	Generic Static Micro-isolators 18.5 × 29.5 cm poly-carbonate	Generic Static Micro-isolators 18.5 × 29.5 cm poly-carbonate	Generic Static Micro-isolators 18.5 × 29.5 cm poly-carbonate	Alternative designs, poly-carbonate	Standard mouse micro-isolator tops 7.75 × 12 in. of poly-carbonate/ polysulfone	Standard mouse micro-isolator tops 7.75 × 12 in. of poly-carbonate/ polysulfone	None	Generic Static Micro-isolators 18.5 × 29.5 cm poly-carbonate
Enrichment	Nestlets, igloos, PVC pipes	Nestlets	None	None	None	None	None	None	None	None	None	None
Illumination												
Light/dark (LD) pattern	14:10	14:10	14:10	14:10	14:10	14:10	14:10	14:10	12:12	12:12	12:12	14:10
Light on:light off	0600 h, 2000 h	0600 h, 2000 h	0600 h, 2000 h	0600 h, 2000 h	0600 h, 2000 h	0600 h, 2000 h	0600 h, 2000 h	0400 h, 1800 h	0600 h, 1800 h	0600 h, 1800 h	0600 h, 1800 h	0600 h, 2000 h
Light-intensity (light phase)	30 FC at 1 m	30 FC at 1 m	30 FC at 1 m	30 FC at 1 m	30 FC at 1 m	30 FC at 1 m	30 FC at 1 m	30 FC at 1 m	35 FC activity chamber, 20 FC elevated plus maze	30 FC Dowel, Porsolt, rotarod, 50 FC sleep	50 FC at 1 m	30 FC at 1 m
Contact person												
Direct animal contact	Caretakers + husbandry technicians	Caretakers + husbandry technicians + experimenter	Caretakers + husbandry technicians + experimenter	Caretakers + husbandry technicians + experimenter	Caretakers + husbandry technicians + experimenter	Caretakers + husbandry technicians + experimenter	Caretakers + husbandry technicians + experimenter	Technician only	Caretakers + husbandry technicians + experimenter	Caretakers + husbandry technicians + experimenter	Caretakers + husbandry technicians + experimenter	Caretakers + husbandry technicians + experimenter
Human presence (time)	Maximum 8 h	Maximum 8 h	Maximum 8 h	Maximum 8 h	Maximum 8 h	Maximum 8 h	Maximum 8 h	Maximum 8 h	8 h	3 h	2 h	Maximum 8 h
Handling method (hands, transfer box, restrainer)	Hands	Hands, denim pockets	Hands	Hands	Hands	Hands	Hands	Hands	Hands	Hands	Hands	Hands
Protection wear												
Gloves, mask, suit/ labcoat, shoes	Gloves, masks (for allergy sufferers), barrier-dedicated scrubs + shoes	Gloves, masks (for allergy sufferers), barrier-dedicated scrubs + shoes	Gloves, masks (for allergy sufferers), barrier-dedicated scrubs + shoes	Gloves, masks (for allergy sufferers), barrier-dedicated scrubs + shoes	Gloves, masks (for allergy sufferers), barrier-dedicated scrubs + shoes	Gloves, masks (for allergy sufferers), barrier-dedicated scrubs + shoes	Gloves, masks (for allergy sufferers), barrier-dedicated scrubs + shoes	Gloves, mask, gown, hairnet, shoe covers	Gloves, masks, disposable gowns, shoe covers, hair bonnets	Gloves, masks, disposable gowns, shoe covers, hair bonnets	Gloves, masks, disposable gowns, shoe covers, hair bonnets	Gloves, masks (for allergy sufferers), barrier-dedicated scrubs + shoes
Food												
Brand, type, % fat, % protein	Irradiated Purina 5053: 5% fat, 20% protein	Irradiated Purina 5053: 5% fat, 20% protein	Harlan Teklad #8640	Harlan Teklad #8640	Harlan Teklad #8640	Harlan Teklad #8640	Harlan Teklad #8640	Harlan Teklad #8640	Harlan Teklad #8640	Harlan Teklad #8640	Harlan Teklad #8640	Harlan Teklad #8640
Water												
pH	Chlorinated 3–5 p.p.m.	Chlorinated 3–5 p.p.m.	Chlorinated 3–5 p.p.m.	Tap water	Tap water	Tap water	Tap water	Tap water	Tap water	Tap water	Tap water	Tap water
Watering system	Automatic: Edstrom	Automatic: Edstrom	Standard cage bottles	Standard cage bottles	Standard cage bottles	Standard cage bottles	Standard cage bottles	Standard cage bottles	Standard cage bottles	Standard cage bottles	Standard cage bottles	Standard cage bottles
Climate												
Ventilated cages (vcs)	Thoren/ Optimice	Thoren/ Optimice	None	None	None	None	None	None	None	None	None	None
Total air/h within vcs	Thoren 50 c.p.h.	Thoren 50 c.p.h.	N/A	N/A	N/A	N/A	N/A	N/A	N/A	N/A	N/A	N/A
	Optimice 20–30 c.p.h.	Optimice 20–30 c.p.h.	N/A	N/A	N/A	N/A	N/A	N/A	N/A	N/A	N/A	N/A
Total air/h	10–20 c.p.h.	10–20 c.p.h.	None	None	None	None	None	10–20 c.p.h.	None	None	None	None
Fresh air/h	100%	100%	None	None	None	None	None	100%	None	None	None	None
Temperature	70 ± 2°F	70 ± 2°F	70 ± 2°F	70 ± 2°F	70 ± 2°F	70 ± 2°F	70 ± 2°F	71 ± 3°F	72 ± 3°F	72 ± 3°F	72 ± 3°F	70 ± 2°F
Humidity	30–70%	30–70%	30–70%	30–70%	40–60%	40–60%	40–60%	22–66%	30–70%	30–70%	30–70%	40–60%
Animals												
Maximum animals/cage	Five adults	Five adults	4	1	1	5	1	Five adults	1	1	1	5
Room specifications												
Acoustic background	Ambient	Ambient	Ambient	N/A	N/A	N/A	N/A	Ambient	Ambient	Ambient	Ambient	N/A
Room space (m^2^)	464 sq. ft. small, 672 sq. ft. large	464 sq. ft. Small, 672 sq. ft. Large	229 sq. ft.	N/A	N/A	N/A	N/A	Approximately 400 sq. ft.	Testing: 198 sq. ft., housing: 256 sq. ft.	Testing: 198 sq. ft., housing: 256 sq. ft.	88 sq. ft.	N/A
Acoustic deprivation	None	Insulation/ dampening	None	N/A	N/A	N/A	N/A	None	None	None	None	N/A
White noise	Caging system	Caging system	Ventilated caging system	N/A	N/A	N/A	N/A	Subzero freezers	Testing equipment	Testing equipment	Testing equipment	N/A
Health and Hygiene												
Parasitology	Quarterly	Quarterly	Quarterly	Quarterly	Quarterly	Quarterly	Quarterly	Bi-anually	As required	As required	As required	Quarterly
Bacteriology	Quarterly	Quarterly	Quarterly	Quarterly	Quarterly	Quarterly	Quarterly	No	As required	As required	As required	Quarterly
Serology	Quarterly	Quarterly	Quarterly	Quarterly	Quarterly	Quarterly	Quarterly	Quarterly	Monthly	Monthly	Monthly	Quarterly
SPF-conditions	Yes	Yes	Yes	No	No	No	No	No	No	No	No	No

To avoid seasonal and other cohort-type effects on the correlation of trait values, each of the batteries was run in parallel with the exception of footshock vocalization and HIC which were collected independently in a related project. For all other test batteries in this study, all strains were tested in all batteries over the same period of several years by the collaborating laboratories. Confounding environmental variation with strain variation was minimized by sampling individuals from the various strains across this multi-year project. Because phenotype analyses can be influenced by fluctuations in laboratory environment that interact with genotype (Chesler *et al.* 2002a,b; Crabbe *et al.* 1999), we recorded laboratory variables such as experimenter, age and test date, which can be matched to records pertaining to the animal colonies and test rooms. These remained largely consistent through the course of this study.

### Neurobehavioral testing procedures

Each mouse was assigned to one and only one battery of testing, and transported by ground in a dedicated SPF van to the appropriate test site. The housing conditions at the testing sites and overall testing protocols are summarized in Tables 1 and 2. Specific methods for each battery of tests were as follows:

#### Adrenal weights

Naïve mice were housed in a fume hood up to 24 h before dissection. On the day of dissection, cages were removed from the hood one at a time. The individual cages were placed on a cart on the far end of the room, separated from the dissection area. Individual mice were removed from each cage one at a time, while their body weight, coat color, sex, birth and histology dates were noted. This was performed calmly so as to minimize the possibility of sympathetic nervous system activation, which might ultimately affect adrenal weights (Ulrich-Lai *et al.* 2006). The individual mice were subject to cervical dislocation and their abdominal cavities opened. Whole kidneys were removed one at a time with the adrenal glands attached. The adrenal glands were identified with the naked eye, as they are lighter flesh-colored compared with the surrounding tissue and are often either attached directly to the kidney or within the connective tissue just anterior to the organ. The adrenal glands of males are generally smaller than those of females. The sample was transferred to the stage of a Zeiss dissecting microscope to facilitate clean dissection of the adrenal gland from the surrounding tissue. Once the adrenal glands had been separated they were weighed on a Mettler Toledo scale to a 10th of a milligram, fixed and stored for subsequent histological analysis.

#### Adult neurogenesis

*BrdU administration and perfusion.* Body weight and coat color were recorded for each animal. Mice were injected with BrdU solution (Sigma, St. Louis, MO, USA; Cat B5002; see below) at 11:00 and were put back in their home cage with a tail mark to indicate test order. Typically, one mouse was injected every 10 min. Fresh BrdU solution 0.5% (5 mg/ml) was prepared before each day's perfusions. BrdU is dissolved in 0.007 N NaOH in 0.9% NaCl. Each mouse was injected with BrdU (50 µg/g body weight or 0.1 ml/10 g body weight) and perfused 1 h after injection. Approximately 5 min before starting the perfusion, the mouse was anaesthetized with Avertin. Mice were perfused transcardially first with 0.1 M phosphate-buffered saline (PBS) and then alcohol–acetic acid solution (1:3, 95% EtOH:acetic acid). Brains were removed and post-fixed in the same fixative overnight with one brain in each vial. The following day, the brains were put into 70% EtOH where they sat, at room temperature, until they were embedded in paraffin. Immediately before embedding, the brains were cut at midline into two hemispheres, dehydrated/defatted in an ethanol–xylene series and placed in 64°C paraffin over night. The following day, brains were transferred twice into fresh paraffin. Brains were then embedded in a mold and cooled for sectioning. Each embedded half brain was serially sectioned in the sagittal plane at 8 µm and every 10th section was mounted on Superfrost/Plus slides. Slides were allowed to dry at 37°C overnight.

*Anti-BrdU immunohistochemistry.* On the first day of BrdU immunostaining for paraffin-embedded 8 µm sections, a xylene–ethanol series is used for deparaffinization. Brains were immersed in distilled water and rinsed in a series of PBS, HCl, 8.C00.404, PBS and hydrogen peroxide PBS. Slides were incubated with mouse anti-BrdU (Sigma, Cat B8434)×200 primary antibody in 5% normal horse serum overnight. On the second day, slides were incubated with horse anti-mouse immunoglobulin G (IgG)×200 secondary antibody for 1 h. Finally, a diaminobenzidine tetrahydrochloride (DAB) reaction is performed using the Vectastain Elite ABC Kit (Vector Laboratories, Burlingame, CA, USA). After development, slide-mounted sections were rapidly dehydrated and cover-slipped.

*Counts of BrdU-labeled cells.* For each animal, adult neurogenesis in the rostral migratory stream (RMS) was evaluated as the number of BrdU-positive cells was calculated for the full length of the structure. In an ideal case, these data were obtained from a single section. However, when necessary, data were retrieved from two sections and very rarely from three sections (the number of sections used for the analysis is recorded). BrdU-positive cells in the RMS were counted using a 40× objective. The RMS length was measured using AnalySIS Opti Version 3.3.776 software (Soft Image System). Only clearly labeled cells were counted in the analysis. The number of BrdU-positive cells per millimeter was calculated by dividing the cell number in each section by its corresponding RMS length. These data were expressed as a total number of BrdU+ cells, and also as the total number of cells divided by the number of sections analyzed to obtain a per section average.

#### Cocaine

*Habituation to a novel environment.* Mice were individually placed into a bank of eight activity chambers (43.2 cm L × 43.2 cm W × 30.4 cm H, ENV-515, Med Associates, St Albans, VT, USA) that contained two sets of 16 photocells placed at 2.5 and 5 cm above the chamber floor. Activity was measured as photocell beam breaks and converted into horizontal distance traveled (cm), and the number of rears was also recorded. Rears were automatically counted when a mouse broke the upper set of photocell beams. In addition, the test chambers were subdivided into a peripheral zone that encompassed a corridor adjacent to each wall that was 7.6 cm wide and central zone (28 cm^2^). Total distance traveled and rears were also separately compiled for both zones. All measures were collected at 15 min intervals during the 1 h test and also expressed as totals over the hour. As an indicator of the distribution of activity, the novelty ratio was calculated as (distance traveled in the periphery/total distance traveled) × 100.

*Locomotor response to saline or cocaine injections.* The same methods, apparatus and dependent measures were used with the exception that the activity chambers were not subdivided into peripheral and central zones. On successive test days, mice were injected (i.p.) with isotonic saline (10 ml/kg) or cocaine (10 mg/kg in isotonic saline at a volume of 10 ml/kg) and immediately placed in the activity chambers. An additional measure of cocaine sensitization was calculated by subtracting total distance traveled after the first cocaine injection from total distance traveled after the second cocaine exposure. Positive values indicated sensitization.

*Conditioned place preference.* Eight chambers were used (ENV-3013, Med Associates). Each chamber (46 cm L × 14 cm W × 20 cm H) was subdivided into a center chamber (10 cm L, painted gray with a solid floor) separated by guillotine doors from two conditioning chambers (18 cm L). One conditioning chamber was painted black and had a wire-mesh floor while the other was painted white with a stainless steel grid floor, in order to provide distinctive visual and tactile cues. Three sets (transmitter and receiver) of infrared photocells were spaced equidistantly along the long wall of the place preference conditioning boxes (2 cm above the floor) in order to record the time spent in each conditioning chamber. Mice received either injections of saline or cocaine [3.2 mg/kg (i.p.) in saline vehicle]. Testing was conducted over 5 days using procedures similar to those of Seale and Carney (1991): *Day 1*—Each mouse was introduced into the middle of the place conditioning apparatus. The guillotine doors were raised and the time spent on each side was automatically recorded during this 20 min baseline session. *Days 2*–*4*—There were a total of three conditioning days that totaled 40 min in duration. The guillotine doors remained closed during conditioning sessions. All mice were injected with saline and placed in the black compartment. After 20 min, each mouse was removed from the apparatus and briefly returned to its home cage. The animals were then injected with cocaine (3.2 mg/kg) and placed into the white compartment for an additional duration of 20 min. *Day 5*—Place preference was evaluated in a 20 min test. Each mouse was introduced into the middle of the apparatus and the guillotine doors were raised. The time spent on each side of the apparatus was recorded. The dependent measures included the time (seconds) spent on the drug- and saline-paired sides at baseline (Day 1) and test (Day 5). In addition, change in preference was calculated as time spent on the drug-paired side at test minus the time spent on the drug-paired side at baseline. Positive numbers indicated an increased preference.

#### Morphine

*Habituation to a novel environment.* Habituation to a novel environment was conducted using the same procedure as in the cocaine tests.

*Locomotion in response to an injection of morphine.* For the test of locomotion in response to an injection of morphine, the methods of Kest *et al.* (2002a,b); and Schulteis *et al.* (1997) were used. Mice received a single injection (i.p.) of morphine sulfate (50 mg/kg in isotonic saline at a volume of 10 ml/kg) and were immediately placed into the activity chambers. Horizontal distance traveled and rearing were recorded in 15 min intervals throughout the 3 h test and also expressed as totals over the 3 h.

*Behavioral (morphine withdrawal) response to an injection of naloxone.* Mice were briefly removed from the activity chambers and injected (i.p.) with naloxone (30 mg/kg in isotonic saline at a volume of 10 ml/kg), and immediately returned to the chambers for an additional 15 min. Horizontal distance traveled was automatically recorded. The effect of naloxone on activity was calculated as distance traveled between 165 and 180 min post-morphine minus total distance traveled following naloxone. In addition, over the 15 min post-injection of naloxone, a trained observer counted the number of jumps, fecal boli and urine puddles that each mouse produced. Jumps were defined as all 4 ft out of contact with the floor of the chamber and the mouse in an upright posture. In addition, between 5 and 10 min post-naloxone, somatic signs of withdrawal intensity were rated by a trained observer similar to the weighted scale of Gellert and Holtzman (1978). This scale consisted of graded ranking (range = 1–3) of wet dog shakes, instances of abdominal contraction, salivation, ptosis and abnormal posture.

#### General behavior

The general behavior phenotyping battery was performed as previously described (Cook *et al.* 2001, 2007). The open field, light–dark and fear conditioning tests were performed using the Hamilton-Kinder SmartFrame system (Hamilton-Kinder, Poway, CA, USA) and test-specific inserts described below.

*Zero-maze.* Briefly, animals were brought into a darkened testing area a minimum of 30 min before testing and allowed to acclimate. The test apparatus is a plexiglass maze placed 108.9 cm off the floor with 40 cm outer diameter and 30 cm inner diameter, and closed arm walls at 28.5 cm H (AccuScan Instruments, Inc., Columbus, OH, USA) were dimly illuminated by 15 W red bulbs suspended above the maze. To begin the test, animals were placed into a closed quadrant of one of the three identical mazes. The test session is 5 min in duration. Once an animal has been tested, it is placed in a holding cage until all animals from the home cage have been tested.

*Open field.* Animals were brought into the testing area a minimum of 30 min (but ideally 45 min to an hour) before testing and allowed to acclimate. The open field session was 20 min in length. To begin the test, individual animals were removed from the cages and placed into the center of an open field apparatus (24.13 cm L × 45.72 cm H). Once an animal had been tested, it was placed in a holding cage until all animals from the home cage completed testing.

*Hot plate.* This test occurs approximately 2–3 h after the completion of the open field test. The lights in the testing area were turned off at least an hour prior to testing and animals were allowed to sit undisturbed in the darkened room. A lamp (15 W bulb) behind the hot plate (Hotplate Analgesia Meter, Model 39, IITC, Inc.) was faced away from the hot plate surface. A mirror was placed behind the hot plate so that the experimenter can observe the animal. The hot plate was maintained at 52°C. The mouse was placed on the center of the hot plate in a smoke gray Plexiglas bottomless cube and the built-in timer started. As soon as the animal elicited a pain response (i.e. paw licking, guarding, shaking or jumping), the timer was immediately stopped and the animal removed from the hot plate surface. If the animal did not show a response within 30 seconds, the test was stopped and the animal was assigned the 30 seconds maximum time as its response latency. Once an animal had been tested, it was placed in a holding cage until all animals from the home cage have been tested.

*Light/dark.* The animals were acclimated to the darkened room for a minimum of 30 min. A lamp, with 15 W bulb was located directly above the light portion of the light/dark box which had total dimensions of 24.13 cm L × 45.72 cm W. To begin the 10-min test, animals were placed in the light half of the box. The guillotine door was then removed to allow the animals to freely move between the two halves of the box. The amount of time spent in the light vs. dark compartment was measured. Once an animal had been tested, it is placed in a holding cage until all animals from the home cage have been tested.

*Startle/pre-pulse inhibition.* The startle and pre-pulse inhibition tests were performed using a Hamilton-Kinder SM100 startle chamber inside a 14 in L × 10.875 in W × 19.5 in H sound-attenuating chamber. Animals were placed in the chamber with a 65 dB background white noise and allowed to habituate. Over an approximately 15 min session, 55 pseudo-random trials were given. A 120 dB white noise burst was used as the acoustic startle stimulus. Pre-pulses were 70, 80 and 85 dB white noise bursts which preceded the startle stimulus by 10 milliseconds. Startle responses to the startle stimulus and to each of the pre-pulse dB levels were measured.

*Fear conditioning.* The first part of fear conditioning (Training) was carried out approximately 1–1/2 to 2 h after the startle and pre-pulse inhibition test. Animals were placed in the fear conditioning chambers (24.13 cm × 22.86 cm, with a grid floor) and allowed to habituate for 2.5 min. Animals were then presented with three pairings of an 85 dB tone and 0.36 mA footshock. The tone was 30 seconds in duration, and the shock was presented during the last 2 seconds of the tone. There was a 2.5 min interval between each of the tone plus shock pairings.

*Contextual conditioning.* On the day following the training session, animals were placed back into the same chambers where they underwent training. During the 6 min session, activity (beam breaks) per 30-second bin was measured and compared with activity during the habituation period on the training day.

*Cued conditioning.* Approximately 2 h later, the behavior of the mice was tested in an altered context. The fear conditioning chambers were altered by placing a gray, square tile over the grid floor, placing a black Plexiglas insert over the walls of the chambers, and attaching a small cup containing orange oil diluted in water in the upper corner of the box. Animals were allowed to explore the altered environment for 2.5 min, after which, the conditioned stimulus (tone) is presented for 2.5 min. Activity (beam breaks) was evaluated in 30-second bins.

*Tail suspension.* All animals were weighed to the nearest one-tenth of a gram prior to tail suspension testing. The body weight for each animal was entered into the Med Associates tail suspension program. On the basis of the body weight, a threshold force of movement for each animal is automatically calculated. Mice were suspended by the tail with generic sports tape attaching them to the transducer. During the 6 min test, force of movement or lack thereof was recorded and reflected the time the animal spent immobile during the test.

#### Ethanol 1

*Overview of test sequence.* Each mouse was singly housed on arrival and was given at least 1 week to acclimate before testing. The testing was carried out over a period of 3 days. Half of the mice were given saline on Day 2 and an ethanol injection (2.25 g/kg) on Day 3. For the other half of the mice, the order of injection was reversed with ethanol on Day 2 and saline on Day 3. On Day 1, all mice were trained on the rotarod, as described below. On Day 2, mice were weighed and given the appropriate injection. Ten minutes after the injection, mice were given a 5-min test in the elevated plus maze followed immediately by a 20-min test in the activity chamber. After the activity chamber, mice were tested on the rotarod and blood was taken to measure blood ethanol concentration (BEC). Mice were then returned to the home cage and animal room until testing the next day. On Day 3, order of testing was identical to the second except that the elevated plus maze was not conducted.

*Rotarod.* For each trial, each mouse was placed on its own segment of the rotarod facing the back wall. The rod was spinning at five revolutions per minute (r.p.m.) at the beginning of the test and accelerated to 25 r.p.m. The mouse remained on the rotarod until it fell off. Both the length of time and the speed of the rotarod when the mouse falls were recorded. On Day 1 (training day), each mouse was given 10 trials on the rotarod and data were recorded. Because the behavior of the mice reached a plateau after five trials, the last three were used to calculate the mean for the training day for both speed and time on rotarod in seconds. On Days 2 and 3, only three trials were given and all were used to compute the mean for each condition (saline vs. ethanol). All mean times were recorded. In addition, several computed measures were also determined: training mean–saline mean (to determine the effects of the injection, repeated exposure and prior tests on the rotarod score), training minus ethanol (to determine the effects of ethanol on motor incoordination) and saline–ethanol (this measure may ultimately not be required depending on whether differences were seen between training and saline).

*Elevated plus maze.* Each mouse was placed in the center of the maze facing an open arm of the plus maze which has been previously described (Hamre *et al.* 2007). Mice were given 5 min to explore the maze. Both the amount of time in the various arms and the number of entries into the arms were recorded. The amount of time provides a measure of the degree of anxiety while the number of entries provides a measure of the activity level and insures that mice entered more than one arm. In addition, the amount of time in the middle of the maze was recorded. The percentage of entries into the open and closed arms were computed and recorded.

*Activity chamber.* Mice were placed in the activity chamber for 20 min on Days 2 and 3 as previously described (Hamre *et al.* 2007). Horizontal distance traveled was recorded in centimeters. Activity was recorded in 5-min bins as well as computed for the total time. Differences between saline and ethanol were computed and recorded.

*Blood ethanol concentration.* A nick was made in the end of the tail and 10–20 ml of blood was drawn from each mouse. Blood was drawn both on Day 2 and Day 3, although it was not saved or analyzed from the saline injected animals. The blood was centrifuged and the BEC determined using the Analox ethanol analyzer (Analox, USA).

#### Ethanol 2

*Sleep/wake analysis.* For the entire period of monitoring, each mouse was placed in its own chamber of a Piezo-electric grid and chamber system (Donohue *et al.* 2008). The Piezo chamber detects movement, and software analysis of respiration rates determine sleep or wake for each mouse. The mice had access to food and water *ad lib* while in the chamber. The room was maintained on a 12:12 light:dark cycle. Mice were placed in the chambers between 0900 and 1000 h on Day 1 and were removed on Day 5 at the same time. Each day, the computer, food and water were checked. Otherwise, the mice remained undisturbed.

*Porsolt forced swim test.* Mice were tested in the Porsolt forced swim test at least 72 h after completion of the sleep analysis. The water in the chamber was heated to 25°C. Each mouse was placed in the chamber for 5 min and videotaped. The water was changed for every third mouse. The behavior of each mouse was scored from the videotapes. The total time immobile as well as the time immobile for the last 3 min were scored and analyzed.

*Dowel test.* Mice were tested for the Dowel test at least 48 h after completion of the Porsolt analysis. At baseline, each mouse was placed on the dowel for a maximum of 2 min. If the mouse remained on the dowel for the entire 2 min, it was removed and injected with 2.0 g/kg of ethanol. Each mouse was then placed back on the dowel for a maximum of 5 min immediately after the injection. The test was repeated 30 min after injection. For all three tests, the length of time until the mouse fell off the dowel was recorded.

#### MDMA

*Locomotor response to MDMA.* On Day 1, all mice were weighed, injected with saline (10 ml/kg, s.c.) and placed in the Open Field Chamber (MED-OFA-510, Med Associates). Activity was recorded for 90 min. On Day 2, mice were habituated to the Open Field chamber for 60 min. They were then injected with saline (10 ml/kg, s.c.) or MDMA (10 mg/kg, s.c.). Activity was recorded for 90 min.

#### Pain

*Hargreaves' paw withdrawal test.* Mice were placed on a 3/16th-in. thick glass floor within small (9 cm L × 5 cm W × 5 cm H) Plexiglas cubicles and allowed a habituation period of 120 min. A focused, high-intensity projector lamp beam (IITC Model 336 Plantar Test and Tail-flick Analgesia Meter, Woodland Hills, CA, USA) was shone from below onto the mid-plantar surface of the hindpaw (Hargreaves *et al.* 1988). The beam was aligned to the mid-plantar surface of the left paw with the projector lamp set to 10% idle intensity (II_10_). The lamp was then switched to 25% active intensity (AI_25_) and the latency to respond with withdrawal of the paw from the light or licking of the paw was recorded using the internal timer. To avoid tissue damage, if no response occurred by 30 seconds, the lamp was returned to the idle intensity and removed from the paw. This process was repeated for all mice in the enclosure, and then migrated to the right hindpaw, following an intra-trial period of at least 5 min. Mice were tested for three to six trials depending on the variance observed on each paw, and the three tightest latencies averaged.

*Hot plate.* After 30 min of habituation to the testing room, mice were placed on a metal surface (IITC Inc., Hotplate Analgesia Meter, Woodland Hills, CA, USA) maintained at 54°C (±0.2°C) (HP_54_) within a transparent Plexiglas cylinder (15 cm D; 22.5 cm H) with Plexiglas lid. The latency to respond with a jump, or hindpaw lick or shake/flutter was measured to the nearest 0.1 s with a stopwatch. Two latencies were recorded per mouse with intra-trial separation of 30 seconds and maximum trial duration of 30 seconds. If no response occurred within 30 seconds, the mouse was removed from the hot plate. The apparatus was thoroughly cleansed with MB-10 (QuipLabs, Wilmington, DE, USA) between each mouse tested.

*Tail withdrawal.* As with the hot plate, mice were allowed 30 min of habituation. Although lightly restrained in a denim pocket, the distal half of the mouse's tail was dipped into a bath of water thermostatically controlled at 47.0°C (±0.1°C) (TW_47_) by Boekel Scientific/Grant Optima Immersion Circulator Model GR150 (Boekel Scientific, Feasterville, PA, USA). Latency to respond to the heat stimulus by vigorous flexion of the tail was measured. Mice received three to five trials separated by 10 seconds, with maximum trial duration of 30 seconds. If no response occurred by 30 seconds, the mouse's tail was removed from the hot water. The last three trials were recorded.

*Tail clip.* As in hot plate and tail withdrawal tests, mice were allowed 30 min of habituation. The enclosure is a Plexiglas-bound arena measuring 13.5 in L × 16 in W × 15 in H. The front of the arena was open and aligned with the leading edge of the table, such that the experimenter could easily restrain and release mice quickly. All mice were lightly restrained in a denim pocket and an alligator clip with a rubber cuff around each jaw (exerting ≈600 g of force) was applied to the tail 1 cm from the base and vertically oriented with respect to the table. The mouse was immediately removed from the holder, and the latency to lick, bite or grab the clip or bring the head within 1 cm of the clip was measured with a stopwatch to the nearest 0.1 second, after which the clip was immediately removed. Each mouse was tested only once with maximum trial duration of 60 seconds. If no response occurs by 60 seconds, the tail clip was removed. The enclosure and clip were thoroughly cleansed with MB-10 (QuipLabs) between each mouse.

*von Frey test.* Mice were allowed 120 min of habituation to the same Plexiglas enclosure (9 cm L × 5 cm W × 5 cm H) as used in Hargreaves' test on a wire-mesh floor (aquarium/vivarium top) instead of a glass floor. For assessment of mechanical sensitivity thresholds, mice were tested with von Frey type nylon monofilaments. A set of eight calibrated von Frey fibers (Stoelting, IL, USA), ranging from 0.067 to 9.33 g of force, were applied to the plantar surface of the hindpaw until they bowed. The threshold force required to elicit withdrawal of the paw (median 50% paw withdrawal) was determined using the up–down method (Chaplan *et al.* 1994). A maximum of nine trials were required for each paw with four trials performed after the first response cross-over. As in the Hargreaves test, trials began with the left hindpaw and then switched to the right after all animals in the enclosure had received the stimulus. An intra-trial period of 150 seconds was used between left and right paws, such that each paw's stimulus was separated by 5 min.

#### Vocalization

*Stress vocalization.* The footshock stimulus to which animals audibly vocalized were assessed following the generation of a mild footshock by Med Associates, Inc. Shock Titration Package for Mice (model ENV-307 W, St Albans, VT, USA). Specifically, on each test day, mice were moved from the mouse colony room to a holding room adjacent to the footshock chambers. Following at least a 25-min wait period (to allow for acclimation to the move), audible vocalization thresholds was assessed. Mice were placed individually in a shock chamber and allowed to adapt to the chamber for 5 min. Each mouse then received a mild footshock via the floor grid every 30 seconds for 500 milliseconds. The intensity of the first footshock was 0.05 mA and each subsequent footshock increased in increments of 0.05 mA until the mouse vocalized as determined by a single technician who performed all assays, positioned within 1 m of the shock chamber. Once the mouse vocalized, the experiment was terminated and the mouse was subsequently removed from the chamber. Each chamber was cleaned between test subjects. To control for experimenter-related variation in audible vocalization detection, the same technician collected every data point and was blind to the subjects' genotype. Naïve mice were held in the adjacent room during the time other subjects were being tested so that they could not hear or otherwise be influenced by the vocalizations of other subjects (Matthews *et al.* 2008).

#### Handling-induced convulsions

Handling-induced convulsions were determined using a modified scoring paradigm from Buck *et al.* (1997). Briefly, each mouse was gently picked up by the tail and spun, if required, to determine a baseline convulsion score. Mice were then injected, i.p., with 4.0 g/kg ethanol (20%) and HIC scores re-determined at 4, 6 and 7 h post-injection. HIC scores were the combined difference scores (i.e. baseline score subtracted from the later scores) for each value following the injection.

### Analysis methods and data access

MouseTrack (Baker *et al.* 2004) and GeneNetwork (Chesler *et al.* 2004; Wang *et al.* 2003) are the two main resources used for data storage, sharing and analysis. MouseTrack serves as the primary data archive and analysis engine for individual mouse data, whereas GeneNetwork serves as the database and analysis engine for QTL mapping, genetic correlation of strain means and integration with other public data from the BXD RI reference population. MouseTrack consists of an ORACLE database and customized sas (version 9.1.3, Cary, NC, USA) client tools for genetic analysis. MouseTrack's RI analysis tools include univariate analysis, box plots of individual strain data, and linear models of sex, treatment and strain effects and interactions, heritability, sub-population effects, and estimation of strain means and strain means by sex for export into GeneNetwork.org. Detailed information about each of the phenotypic values and protocols used to generate them are also accessible from MouseTrack. The MouseTrack tool also performs individual outlier detection, multi-variate outlier detection and distributional evaluation including displays of the phenotypic distributions within strain. These tools can be used to identify phenotypically extreme strains for advanced study. In the present report, outlier detection tools were used exclusively for quality assurance. When extreme univariate outliers are detected (> 5 SD), possible data entry errors and other traceable sources of outliers were considered. If none could be found, the outliers were retained in subsequent analyses and in our submission to GeneNetwork.org.

### Strain effects and sex differences

Sex, strain and strain × sex interaction effects were tested using non-sequential sums of squares estimation in a general linear model. The model used for testing these effects was
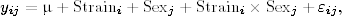
(1)

where *y*_*ij*_is the phenotype being measured for Strain_*i*_ and Sex_*j*_. In addition to testing these effects, we estimated the magnitude of strain, sex and strain × sex effects by estimating the intra-class correlation, a partial ω^2^, for each effect per trait. Partial ω^2^ was estimated as the proportion of variance accounted for by the main or interaction effects relative to the total phenotypic variance. All variance components were estimated using the REML option of SAS PROC VARCOMP. This method can be biased due to departures from normality, a common phenomenon for behavioral traits. The percent variance accounted for by strain is considered by some to be an estimate of broad-sense heritability (Hirsch 1967; Lynch & Walsh 1998) for clones, and is formulated as the strain intra-class correlation:

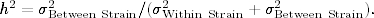
(2)

Standard errors were obtained using an adjustment for unbalanced data (Swiger *et al.* 1964). This calculation was performed for the two sexes separately, and for the data combining both sexes. It should be noted that this measure provides an estimate of the resemblance among relatives in a population in which segregation had occurred and does not reflect the transmission of genetic material in a randomly mating population. It is nonetheless an indication of suitability and plausibility of genetic analysis for a given phenotype.

### Sub-population effects

A limited number of systematic differences exist in the genotypes of the old and new BXD RI strains such that loci have been identified that segregate in only one of the two sub-populations (Shifman *et al.* 2006). To test for global phenotypic effects of these differences, we use the nested model,
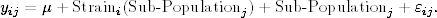
(3)

An alternate *F*-test is applied. The *F* ratio is MS_Sub- Population_/ MS_Strain(Sub- Population)_. This test is used because the individual mice in the study are not independent, but rather are replicates within a strain. Therefore, we use the appropriate error term and degrees of freedom for the random effect of strain nested within the sub-populations. To account for multiple testing, the false discovery rate (FDR) was controlled using *q*-value estimation for the sex and strain main effects and sex × strain interaction effects. The *R*/*q*-value software developed by Storey (2002) was used for the analyses. A *q*-value threshold of 0.05 was used to identify significant results. Quantitative trait locus mapping was also performed separately for sub-populations.

### Genetic analysis in gene network

GeneNetwork serves as a database and analysis engine for QTL mapping and genetic correlations among strain phenotypic means obtained in the BXD RI reference population. Strain means were computed in MouseTrack by sex and other cofactors. For pooled sexes, the mean values were the least-squares means with strain, sex and strain × sex in the model. Male and female strain means for each phenotyping battery were exported from MouseTrack for submission to GeneNetwork.org. This enables gene expression correlation and interval mapping, candidate gene searches and multi-trait analyses.

Each exported dataset was subject to an interval mapping analysis, which uses GeneNetwork's embedded MapManager software (Manly *et al.* 2001) to perform Haley–Knott regression. Empirical *P*-values were derived using 1000 permutations using the incorporated permutation feature of WebQTL. The peak of each statistically significant (*P*-value < 0.05) or suggestive (*P*-value < 0.63) (Lander & Kruglyak 1995) QTL was determined based on empirical *P*-values (Doerge & Churchill 1996). A one-LOD drop-off was used to determine the QTL confidence interval about each peak. Positional candidates residing within an one-LOD drop from the peak of each statistically significant and suggestive QTL were identified. Trait data were correlated against the following GeneNetwork gene expression tissue databases: whole brain [INIA Brain mRNA 430 (June 2006) RMA (Peirce *et al.* 2006)], neocortex [HQF BXD NeoCortex ILM6v1.1 (February 2008) RankInv (Gaglani *et al.* 2009)], striatum [HBP Rosen Striatum M430V2 (April 2005) RMA (Rosen *et al.* 2009)], cerebellum [SJUT Cerebellum mRNA M430 (March 2005) RMA (Chesler *et al.* 2005)] and hippocampus [Hippocampus Consortium M430V2 (June 2006) RMA (Kempermann *et al.* 2006)]. Lists of genes were generated based on their correlation to each phenotype using a correlation *P*-value <0.001.

### Multi-trait QTL analysis in the expanded BXD RI lines

Multi-trait QTL analysis can be performed by extracting common underlying factors from multiple behavioral phenotypes and generating strain-specific factor scores. The underlying hypothesis of this type of analysis is that behavioral measures for stress, anxiety, pain and addiction to drugs of abuse are under common genetic control and should share some degree of correlation. Brigman *et al.* (2009) perform a similar decomposition of anxiety and fear behavior in BXD RI mice. This approach to multi-trait QTL mapping has been undertaken in a study by Trullas and Skolnick (1993), wherein factor analysis was used to report that elevated plus maze behavior predicts anxiety-like behavior, and more recently by Henderson *et al.* (2004), who performed QTL mapping on multiple phenotypic assays of anxiety-like behavior. The value of performing such studies in the BXD RI population is that the data can be expanded indefinitely with additional independent phenotypic profiling which adds depth and detail to the multi-dimensional analysis.

As the phenotypic data contained missing observations, any strain with more than 25% of trait data missing was removed from this analysis. This resulted in the elimination of 32 of the 95 BXD strains, giving us an effective sample size of 63 BXD strains. The resultant data set was subjected to column mean imputation in order to fill in the missing trait values, given that the data were missing at random, i.e. not missing over all measures within a battery. Another issue that was encountered during this analysis was that of dimensionality. The dataset used in the factor analysis had more variables/traits (*p*) than observations/strains (*n*) (i.e. *n < p*). This results in incorrect estimation of the covariance matrix and thereby leads to singularity of the estimated covariance matrix. This issue was addressed by using the James–Stein-type shrinkage estimator (Schafer & Strimmer 2005) of the covariance matrix. *R* packages e1071 (for missing data imputation), corpcor (for covariance shrinkage), nFACTOR and factanal were used for the purposes of factor analysis. Factor loadings were analyzed to obtain factor interpretations. Factor scores were obtained for all interpreted latent factors. Quantitative trait locus mapping was performed on these latent factors to identify common genetic drivers of variability in factor scores.

### Combinatorial analysis of the gene–phenotype associations

Each set of gene expression correlates with *P*-value < 0.001 for gene–phenotype association was subject to combinatorial analysis to identify those genes that were directly correlated to multiple phenotypes. Positional candidates defined as those which reside within an one-LOD confidence interval of significant (*P*-value < 0.05) or suggestive (*P*-value < 0.63) QTL. The top 5 and 10 percent of the highly connected genes were analyzed for over-representation of Kyoto Encyclopedia of Genes and Genomes (KEGG) pathways (Kanehisa & Goto 2000) using the analysis tool, WebGestalt (Zhang *et al.* 2005), which performs a hyper-geometric test for the similarity of the list of highly connected genes and the members of curated pathways in the KEGG database. The resulting list of enriched categories of genes represents those processes, functions and molecular classes that are most involved in genetic variation in behavior.

### Accessing these data

Primary data generated from this behavioral phenotyping project are stored in the MouseTrack system (https://mouse.ornl.gov/mousetrack/). The strain means for each trait by sex were deposited into GeneNetwork.org, and the positional candidates and coexpressed gene lists were stored in a database and tool set called ‘ontological discovery environment (ODE)’ (Baker *et al.* 2009) where they may be integrated with other genomic data sets. The entire analysis path from MouseTrack through GeneNetwork through ODE can be repeated from the primary data in MouseTrack for any individual field. Accession numbers for each trait are listed in Supplementary information, Table S2.

## Results

### Strain effects, sex differences and interactions

Generalized linear models were used to test for sex and strain main effects, and sex × strain interaction effects (Table 3, Supplementary information, Table S1). False discovery rate analysis was used to control the family-wise error rate at 0.05 and showed significant sex effects for 97 of the 257 measures with five expected false positives, significant strain effects for all 257 measures, and significant sex × strain interactions for 144 of the 257 measures with seven expected false positives. In the event that there was no trend toward a significant sex difference or interaction, male and female data were combined for subsequent QTL mapping and genetic correlations.

**Table 3:** Summary of strain and sex effects

	*P*-values for main and interaction effects			Effect sizes (partial ω^2^)			Maximum trait value		Minimum trait value		Variance accounted for by strain in each sex	
Battery and trait	Strain	Sex	Strain × Sex	Strain	Sex	Strain × Sex	♀	♂	♀	♂	♀ (% ± SE)	♂ (% ± SE)
Adrenals												
Left adrenal weight	9.09E–45	1.06E–106	1.20E–10	0.22	0.47	0.05	3.48	2.48	1.60	1.28	0.58 ± 0.05	0.54 ± 0.05
Neurogenesis												
BrdU-labeled adult RMS neurons 1 h post-BrdU	2.81E–04	4.39E–01	4.09E–02	0.20	0.00	0.07	157.33	142.00	59.00	63.00	0.36 ± 0.07	0.12 ± 0.03
Cocaine												
Open field rears 0–15 min post-first cocaine	1.16E–65	6.75E–05	1.43E–03	0.29	0.01	0.02	709.33	680.20	53.53	132.14	0.35 ± 0.04	0.34 ± 0.04
Open field locomotion (cm) 0–15 min post-second cocaine	3.46E–72	1.10E–01	2.24E–01	0.33	0.00	0.01	14 368.75	15 959.85	1583.05	2114.40	0.42 ± 0.05	0.30 ± 0.04
Open field rears 0–15 min post-second cocaine	5.60E–79	1.81E–02	3.61E–05	0.33	0.00	0.03	891.33	834.00	65.42	141.44	0.41 ± 0.05	0.37 ± 0.04
Open field novel total rears in the center	5.63E–82	5.93E–03	6.94E–04	0.40	0.00	0.02	981.80	977.00	6.42	36.72	0.33 ± 0.04	0.41 ± 0.05
Cocaine total locomotion (cm/h)	1.95E–77	1.76E–04	6.58E–01	0.34	0.00	0.00	39 906.11	38 282.20	3236.91	5460.40	0.41 ± 0.05	0.33 ± 0.04
Cocaine open field total rears	9.70E–90	1.86E–07	4.20E–03	0.36	0.01	0.02	2610.67	2946.93	189.88	548.67	0.45 ± 0.05	0.39 ± 0.05
Novel open field–periphery locomotion/total locomotion	1.68E–89	5.94E–05	1.13E–01	0.38	0.01	0.01	79.16	74.62	38.13	40.50	0.38 ± 0.04	0.41 ± 0.05
Novel open field–total locomotion (cm/h)	2.40E–108	4.74E–01	3.27E–02	0.45	0.00	0.01	23 943.62	21 281.60	2645.11	3587.94	0.41 ± 0.05	0.48 ± 0.05
Novel open field–total rears	1.08E–126	8.32E–03	2.51E–05	0.49	0.00	0.02	1943.64	2093.93	140.37	302.50	0.45 ± 0.05	0.54 ± 0.05
Cocaine sensitization–total locomotion (cm/h)	1.15E–82	1.31E–07	2.60E–01	0.35	0.01	0.01	42 524.62	51 737.39	4952.02	5059.96	0.46 ± 0.05	0.32 ± 0.04
Cocaine sensitization–total rears	6.48E–99	6.04E–08	3.83E–06	0.38	0.01	0.03	2979.56	3320.20	263.00	564.33	0.46 ± 0.05	0.42 ± 0.05
Cocaine sensitization–total locomotion (cm/h) minus cocaine total locomotion (cm/h)	2.00E–06	1.80E–03	3.43E–01	0.05	0.00	0.00	8926.98	22 425.55	−2086.00	−2434.76	0.12 ± 0.02	0.04 ± 0.01
Open field total rears post-saline	8.79E–98	6.06E–02	1.37E–05	0.38	0.00	0.01	2107.00	2005.47	82.11	157.56	0.46 ± 0.05	0.40 ± 0.05
Cocaine-conditioned place preference	2.77E–08	9.85E–01	8.38E–04	0.08	0.00	0.04	28.08	25.90	−7.70	−15.29	0.11 ± 0.02	0.11 ± 0.02
Ethanol 1												
Difference in distance traveled (cm) 0–5 min (saline–ethanol)	3.88E–02	5.31E–02	9.81E–01	0.17	0.02	0.00	1592.94	1127.97	−489.25	−843.42	0.20 ± 0.03	0.17 ± 0.02
Distance traveled (cm) 0–5 min after ethanol	9.08E–07	6.84E–02	6.73E–01	0.26	0.02	0.02	2513.60	1827.55	16.92	78.65	0.29 ± 0.06	0.23 ± 0.04
Distance traveled (cm) 0–5 min after saline	2.43E–07	3.20E–01	2.54E–01	0.29	0.00	0.03	1234.00	1263.15	59.56	202.68	0.22 ± 0.05	0.33 ± 0.05
Difference in total distance traveled (cm) (saline–ethanol)	1.03E–03	7.54E–02	7.71E–01	0.20	0.03	0.05	5333.16	3454.36	−1522.22	−4732.01	0.06 ± 0.02	0.19 ± 0.04
Total distance traveled (cm) post-ethanol	5.96E–06	1.16E–01	6.58E–01	0.24	0.02	0.01	7660.92	6031.06	162.18	391.81	0.18 ± 0.04	0.25 ± 0.05
Total distance traveled (cm) post-saline	1.03E–04	4.65E–01	1.37E–01	0.29	0.00	0.12	3518.28	5123.82	439.17	391.81	0.11 ± 0.03	0.30 ± 0.05
Blood ethanol concentration (mg/dl)	8.76E–11	7.68E–01	8.72E–14	0.13	0.00	0.20	350.00	349.70	130.85	145.15	0.70 ± 0.06	0.45 ± 0.06
Percentage of entries into open arms of plus maze	9.75E–01	3.45E–01	7.80E–01	0.01	0.00	0.01	74.39	81.50	2.54	11.79	0.05 ± 0.01	0 ± 0
Percentage of time in open arms of plus maze	6.67E–01	5.55E–01	9.74E–01	0.04	0.00	0.00	78.24	80.38	0.80	4.19	0.03 ± 0.01	0 ± 0
Difference in time on rotarod (training–ethanol)	2.46E–04	7.06E–01	8.73E–01	0.09	0.00	0.00	55.98	28.52	−33.47	−15.75	0.29 ± 0.06	0.02 ± 0
Mean time on rotarod following ethanol	1.75E–11	1.91E–01	1.58E–01	0.25	0.01	0.01	57.89	68.80	10.25	11.55	0.37 ± 0.06	0.44 ± 0.06
Difference in time on rotarod (saline–ethanol)	4.16E–02	4.85E–01	2.18E–01	0.02	0.00	0.00	26.40	31.25	−46.10	−24.38	0.20 ± 0.04	0.08 ± 0.02
Mean time on rotarod following saline	1.73E–11	5.57E–01	7.77E–01	0.23	0.00	0.00	91.11	68.13	12.07	14.68	0.47 ± 0.07	0.32 ± 0.05
Difference in time on rotarod (training–saline)	2.46E–05	2.25E–01	5.37E–01	0.11	0.00	0.00	50.98	31.71	−24.88	−17.75	0.39 ± 0.06	0.11 ± 0.02
Mean time on rotarod during training	2.25E–20	9.19E–01	8.67E–02	0.38	0.00	0.01	65.00	74.09	2.82	9.35	0.47 ± 0.07	0.57 ± 0.06
General behavior												
Thermal nociception hot plate latency	2.18E–16	4.40E–01	4.34E–01	0.14	0.00	0.00	12.82	12.53	5.70	4.72	0.17 ± 0.03	0.13 ± 0.02
Light–dark box total distance traveled in both compartments	3.55E–112	2.66E–01	3.58E–02	0.42	0.00	0.01	1538.00	2285.50	396.00	394.56	0.45 ± 0.05	0.50 ± 0.05
Light–dark box % distance traveled in light compartment	4.41E–26	1.10E–03	3.72E–01	0.15	0.01	0.00	52.57	58.44	17.74	19.74	0.21 ± 0.03	0.15 ± 0.02
Light–dark box % time in light	5.35E–26	8.84E–04	1.18E–01	0.15	0.01	0.01	63.39	73.08	17.24	13.12	0.22 ± 0.03	0.15 ± 0.03
Light–dark box transitions	1.91E–97	6.83E–01	1.89E–01	0.38	0.00	0.00	60.43	64.50	8.50	10.00	0.39 ± 0.05	0.40 ± 0.05
Activity in altered context–fear conditioning apparatus	1.63E–93	2.26E–01	2.56E–03	0.37	0.00	0.02	51.83	54.00	14.88	16.60	0.43 ± 0.05	0.42 ± 0.05
Baseline activity in fear conditioning apparatus	3.43E–90	7.08E–01	3.61E–03	0.36	0.00	0.02	44.82	65.13	13.38	14.54	0.41 ± 0.05	0.41 ± 0.05
Contextual activity in fear conditioning apparatus	5.67E–43	8.41E–01	1.52E–04	0.21	0.00	0.03	35.99	39.55	5.15	4.41	0.27 ± 0.04	0.25 ± 0.04
Activity in altered context during cue presentation	8.17E–78	5.80E–04	6.37E–02	0.33	0.01	0.01	16.98	25.25	1.33	1.69	0.33 ± 0.04	0.37 ± 0.04
Suppression of activity in altered context	4.97E–34	1.24E–03	1.50E–01	0.21	0.01	0.01	0.52	0.78	0.01	0.07	0.21 ± 0.03	0.25 ± 0.04
Activity during first tone shock pairing	9.02E–71	8.07E–01	1.07E–02	0.31	0.00	0.02	46.03	73.00	13.20	16.50	0.28 ± 0.04	0.38 ± 0.05
Activity in 30 s interval post-first tone shock pairing	2.64E–65	4.02E–04	5.13E–01	0.30	0.01	0.00	24.25	31.50	1.31	2.83	0.32 ± 0.04	0.33 ± 0.04
Cue conditioning–activity suppression after third tone/shock pairing	4.23E–10	5.16E–05	6.43E–01	0.07	0.01	0.00	0.82	0.91	0.09	0.13	0.07 ± 0.01	0.10 ± 0.02
Open field–percentage center distance	1.08E–77	3.67E–07	1.95E–01	0.32	0.01	0.00	40.98	47.59	14.16	14.32	0.34 ± 0.04	0.38 ± 0.05
Open field–habituation ratio (first:last intervals)	3.03E–26	2.21E–01	6.47E–02	0.15	0.00	0.01	0.74	0.65	0.32	0.36	0.22 ± 0.03	0.17 ± 0.03
Open field–total rears 0–5 min	1.18E–71	1.41E–01	5.35E–04	0.30	0.00	0.02	61.80	62.67	0.60	2.86	0.36 ± 0.05	0.36 ± 0.04
Open field–total number of rears	6.73E–72	7.24E–01	4.96E–03	0.31	0.00	0.02	235.63	203.80	22.20	32.67	0.35 ± 0.05	0.36 ± 0.04
Open field–total distance traveled	1.66E–108	1.75E–01	9.14E–02	0.41	0.00	0.01	3304.85	5036.50	612.82	853.50	0.49 ± 0.05	0.43 ± 0.05
Background startle response	5.31E–50	4.15E–05	2.08E–29	0.24	0.01	0.15	0.22	0.74	0.03	0.04	0.28 ± 0.04	0.58 ± 0.05
Maximum startle response to 80 dB	3.05E–118	1.30E–04	2.27E–04	0.46	0.01	0.02	2.73	2.56	0.09	0.10	0.53 ± 0.05	0.57 ± 0.05
Pre-pulse inhibition at 80 dB	7.78E–62	1.08E–01	1.60E–02	0.30	0.00	0.02	83.41	79.07	14.62	14.59	0.38 ± 0.05	0.36 ± 0.04
Acoustic startle 80 dB % maximum startle response	7.78E–62	1.08E–01	1.60E–02	0.30	0.00	0.02	85.38	85.41	16.59	20.93	0.38 ± 0.05	0.36 ± 0.04
Tail suspension test–time below threshold	1.50E–16	1.82E–01	2.43E–01	0.32	0.00	0.01	288.83	280.00	16.00	47.25	0.36 ± 0.07	0.42 ± 0.07
Zero-maze–total activity count; beam breaks	3.54E–55	8.97E–01	2.62E–01	0.32	0.00	0.00	834.00	885.50	350.75	302.86	0.34 ± 0.05	0.41 ± 0.05
Zero-maze–total entries in open quadrants	3.04E–32	9.97E–02	9.40E–01	0.23	0.00	0.00	136.80	138.90	25.00	16.60	0.23 ± 0.04	0.24 ± 0.04
Zero-maze–latency to enter an open quadrant	1.00E–19	4.88E–01	1.70E–02	0.16	0.00	0.02	126.30	91.13	4.02	0.45	0.33 ± 0.04	0.14 ± 0.02
Zero-maze–percentage open time	1.27E–34	8.66E–01	5.74E–01	0.24	0.00	0.00	36.13	44.47	2.87	1.98	0.28 ± 0.04	0.26 ± 0.04
Handling												
HICs 4 h after ethanol	3.83E–16	9.99E–01	1.00E+00	0.32	0.00	0.03	3.00	2.83	0.00	0.00	0.19 ± 0.05	0.32 ± 0.06
HIC baseline	6.22E–06	2.90E–01	1.63E–01	0.20	0.00	0.04	2.00	2.67	0.00	0.00	0.15 ± 0.04	0.18 ± 0.04
HIC score	1.77E–11	1.30E–02	1.46E–04	0.23	0.00	0.08	5.50	8.00	0.00	0.00	0.19 ± 0.05	0.49 ± 0.08
MDMA												
Locomotor response–10 mg/kg MDMA	3.16E–03	5.97E–01	7.85E–01	0.71	0.00	0.00	36 001.50	16 648.00	217.50	276.67	0.58 ± 0.22	0.57 ± 0.15
Locomotor activity Day 1	1.06E–01	9.11E–01	6.71E–01	0.50	0.00	0.01	16 310.00	10 970.00	1678.00	818.00	0.51 ± 0.23	0.28 ± 0.12
Locomotor activity after second saline treatment	1.96E–01	3.72E–01	4.91E–01	0.59	0.00	0.03	7598.50	3311.00	137.50	166.00	0.47 ± 0.23	0.24 ± 0.11
Morphine												
Morphine distance (cm) traveled 0–15 min	1.51E–32	3.73E–02	1.61E–01	0.39	0.01	0.02	12 905.20	9172.61	169.92	314.22	0.52 ± 0.06	0.46 ± 0.06
Morphine response–abdominal constriction severity	3.02E–01	4.67E–01	1.93E–02	0.00	0.00	0.00	0.67	0.33	0.00	0.00	0.28 ± 0.05	0 ± 0
Morphine response–defecation	7.05E–08	9.61E–01	2.24E–02	0.17	0.00	0.02	6.00	4.17	0.00	0.00	0.16 ± 0.03	0.26 ± 0.04
Morphine number of jumps	4.44E–03	8.03E–01	8.29E–02	0.15	0.00	0.13	32.20	9.00	0.00	0.00	0.19 ± 0.03	0.13 ± 0.03
Morphine open field total distance (cm) traveled	1.94E–43	8.22E–01	9.44E–02	0.57	0.00	0.02	149 418	171 020	1033.67	1426.82	0.55 ± 0.06	0.62 ± 0.05
Change in distance traveled morphine–naloxone	5.88E–21	1.59E–01	2.31E–01	0.32	0.00	0.00	7186.49	9028.44	−2931.08	−1557.85	0.31 ± 0.05	0.43 ± 0.06
Morphine total vertical activity counts	1.64E–31	4.30E–01	3.75E–01	0.49	0.00	0.02	8838.25	9272.50	123.00	22.00	0.42 ± 0.06	0.54 ± 0.06
Open field novel total locomotion (cm/h) in the center	1.41E–38	4.53E–02	1.70E–02	0.45	0.00	0.02	20 079.97	23 093.42	4411.77	2843.86	0.53 ± 0.06	0.60 ± 0.05
Open field novel total rears in the center	7.17E–40	3.25E–01	1.06E–01	0.52	0.01	0.01	2075.50	1899.00	183.00	310.00	0.55 ± 0.06	0.57 ± 0.06
Naloxone-induced morphine withdrawal distance traveled in 15 min	2.38E–20	4.03E–01	5.20E–02	0.32	0.00	0.02	3543.69	2379.65	539.69	370.29	0.45 ± 0.06	0.34 ± 0.05
Naloxone-induced morphine withdrawal total rears in 15 min	1.81E–17	5.91E–03	9.21E–03	0.24	0.00	0.06	201.00	225.75	9.33	16.33	0.38 ± 0.05	0.37 ± 0.05
Morphine withdrawal–postural effects	4.19E–01	3.35E–01	3.20E–01	0.04	0.00	0.01	1.20	1.25	0.00	0.00	0.11 ± 0.02	0.02 ± 0.01
Morphine–severity of ptosis	2.10E–16	2.41E–02	1.85E–02	0.33	0.00	0.03	2.00	2.67	0.00	0.00	0.27 ± 0.05	0.39 ± 0.05
Morphine–salivation	3.21E–11	8.48E–02	5.67E–01	0.24	0.00	0.00	2.17	1.83	0.00	0.00	0.18 ± 0.03	0.30 ± 0.05
Morphine–wet dog shakes	3.86E–19	4.10E–01	5.42E–02	0.31	0.00	0.02	2.67	3.00	0.00	0.00	0.47 ± 0.06	0.29 ± 0.05
Nociception												
Thermal nociception Hargreaves' test	2.35E–11	1.92E–06	6.00E–02	0.13	0.02	0.02	20.95	23.78	11.73	13.56	0.21 ± 0.03	0.16 ± 0.03
Thermal nociception hot plate two trial average	3.60E–31	3.42E–01	9.52E–03	0.27	0.00	0.03	18.05	16.53	9.13	8.75	0.32 ± 0.05	0.28 ± 0.04
Mechanical nociception–tail clip test	1.85E–30	4.32E–02	2.99E–02	0.26	0.00	0.02	59.13	60.00	23.91	22.36	0.27 ± 0.04	0.32 ± 0.04
Thermal nociception tail withdrawal test	4.15E–19	8.31E–06	2.77E–01	0.18	0.02	0.01	21.75	28.13	11.20	13.29	0.18 ± 0.03	0.24 ± 0.04
Mechanical sensitivity–von Frey threshold	2.17E–17	2.87E–02	1.61E–01	0.17	0.00	0.01	4.45	4.48	4.14	4.17	0.23 ± 0.04	0.21 ± 0.03
Ethanol 2												
Dowel test–time on dowel immediately post-ethanol (s)	4.56E–03	4.33E–02	2.89E–02	0.08	0.00	0.03	163.33	210.00	31.00	5.00	0.04 ± 0.01	0.20 ± 0.04
Dowel test–time on dowel 30 min post-ethanol injection (s)	5.03E–14	1.96E–03	2.21E–04	0.33	0.01	0.06	287.00	223.20	1.60	0.00	0.21 ± 0.04	0.53 ± 0.06
Dowel test–time on dowel at baseline (s)	4.89E–05	6.66E–02	8.30E–01	0.12	0.01	0.00	120.00	120.00	83.00	51.80	0.05 ± 0.01	0.19 ± 0.03
Porsolt time immobile (s)	3.41E–39	1.35E–02	3.01E–03	0.51	0.00	0.03	174.80	223.50	0.25	0.00	0.52 ± 0.06	0.60 ± 0.05
Stress vocalization												
Vocalization threshold–shock intensity (mA)	2.89E–24	3.20E–03	7.21E–05	0.14	0.00	0.01	1.06	0.70	0.16	0.19	0.41 ± 0.05	0.30 ± 0.04

**Figure 1:**
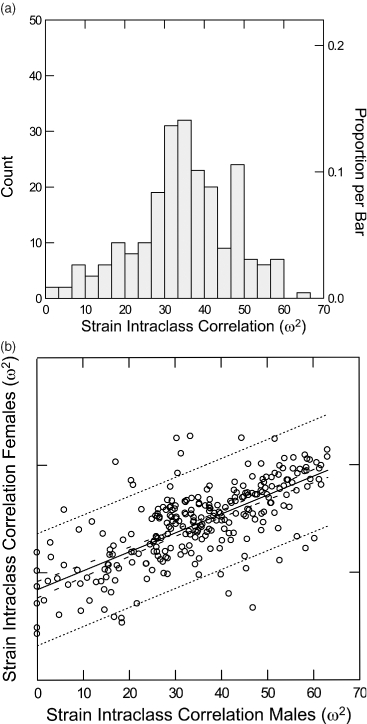
**Strain intra-class correlations for all measures.** (a) Frequency histogram of strain intra-class correlations for both sexes combined. A majority of the behavioral measures (146 of 257) have ω^2^ greater than 30% making them amenable to QTL mapping. (b) Scatter plot of strain intra-class correlations for males and females. Dotted lines are 95% upper and lower prediction intervals for the relationship between these values. Dashed lines are the corresponding 95% upper and lower confidence intervals.

Effect sizes were estimated as partial ω^2^ for the main effects of strain and sex, and for the sex × strain interaction (Table 3). Consistent with statistically significant strain effects, 146 measures under consideration had large effect sizes (ω^2^ > 0.30), 93 had intermediate effect sizes (0.30 *> ω*^2^ > 0.10) and 19 had small effect sizes (ω^2^ < 0.10) (Fig. 1a). Locomotor activity in a variety of apparatus and under different drug exposure conditions typically had large strain effect sizes, consistent with the previous reports (Wahlsten *et al.* 2006). Other traits with large effect sizes include acoustic startle response, pre-pulse inhibition, morphine withdrawal and alcohol withdrawal. Intermediate effect sizes were observed for nociception-related traits, morphine side-effects, ethanol-induced ataxia, baseline HIC, blood ethanol concentration, cocaine-conditioned place preference and anxiety. Only a small number of measures had effect sizes below 0.1. These were most often measures derived from linear combinations of two measures, for which the expected variance of the derived scores is at least four times the variance of either measure alone (Johnson & Wichern 1998). The 30% effect size is a conventional guide for traits amenable to genetic analysis.

The proportion of variance accounted for by strain varies between the sexes for many traits. For some traits, females have a higher proportion of non-genetic variation and for others males exhibit more non-genetic variation (Fig. 1b). Across test batteries, except for adrenal weights, the main effect of strain consistently accounts for most of the variation. The partial ω^2^ for the main effects of sex were considerably lower than the strain main effects, except for left and right adrenal weight measures (Table 3). However, sex × strain interaction effects were of greater magnitude than the main effects of sex, though not as large as strain effects.

### Comparison of new vs. old BXD RI strains

In our comparison of historical strains with the newly expanded BXD lines (BXD Sub-population effects), we evaluated differences among the three groups of BXD RI lines tested simultaneously for all 257 measures. These were BXD 1–32 (Taylor *et al.* 1977), BXD 33–42 (Taylor *et al.* 1999) and BXD 43–100 (Peirce *et al.* 2004). Only the adrenal weight measures show any significant differences among the three groups of strains for the left (*P*-value = 3.23E–04) and right (*P*-value = 1.99 E–04) adrenal weights, when sup-population means are considered. Below we address the issue of mapping in the combined sub-populations.

### Single-trait QTL analysis in the expanded BXD RI lines

**Figure 2:**
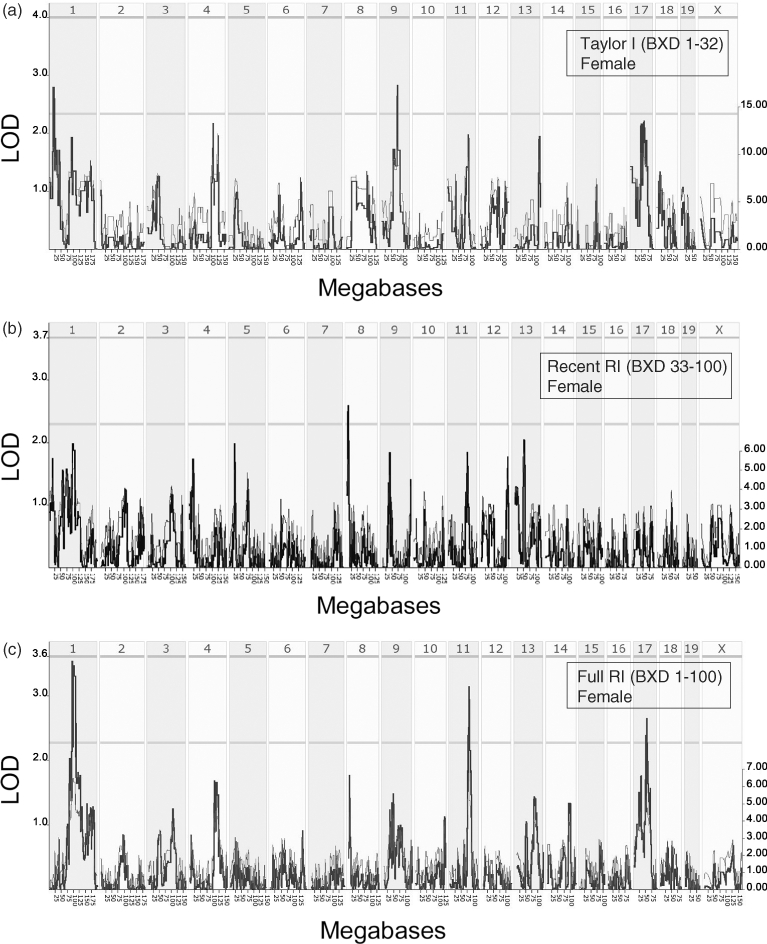

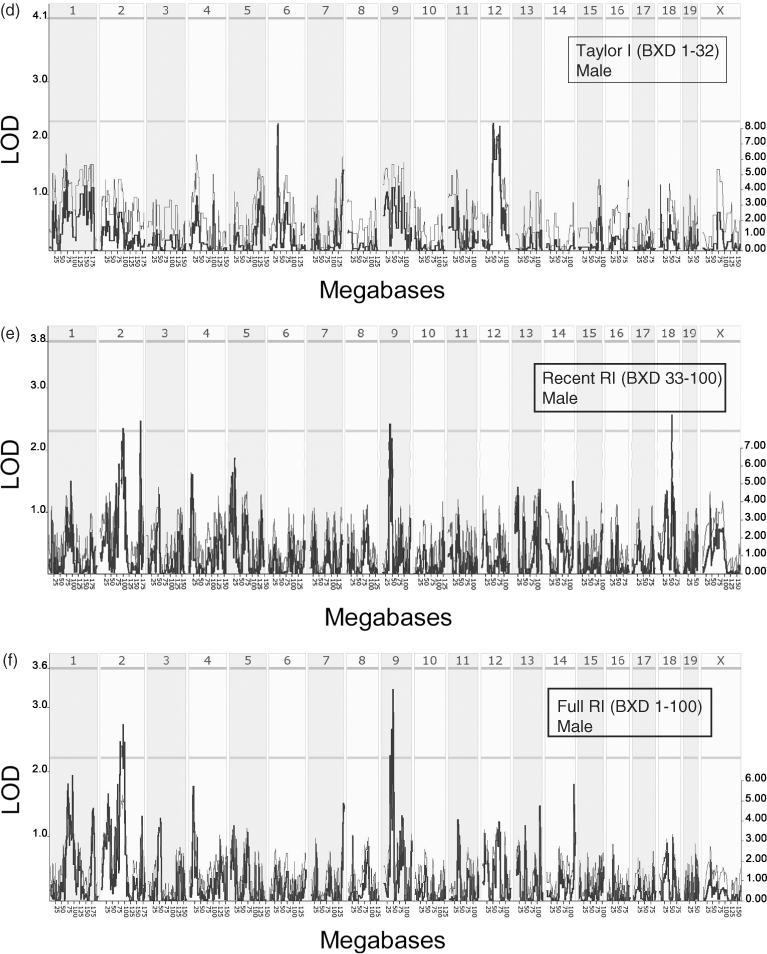
**Quantitative trait locus analysis of mechanical nociception in the original and recent BXD RI panels: QTL analysis of a single-trait, the latency to respond to a plastic-coated smooth alligator clip placed on the tail was performed across three BXD panels, namely, Taylor I (BXD 1**–**32), Recent BXD RI (BXD 33**–**100) and Full BXD RI (BXD 1**–**100).** In females, suggestive QTL were detected on Chr 1 and 9 for Taylor I (a), Chr 8 for the Recent BXD RI (BXD 33–100) (b) and Chr 1, 11 and 17 for the Full RI Lines (c). Mapping of tail clip latency in males showed no suggestive QTL for Taylor I (d), suggestive QTL on Chr 2, 9 and 18 for Recent BXD RI (BXD 33–100) (e), and two suggestive QTL on Chr 2 and 9 for the Full RI panel (f).

**Figure 3:**
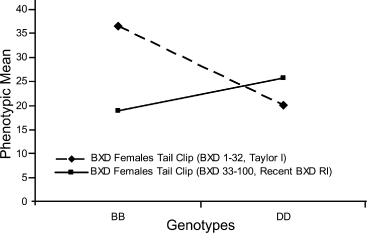
**Effect plot of marker gnf01.018.340 (Chr 1 at 21.43 Mb) in Taylor I (BXD 1**–**32) and Recent RI (BXD 33**–**100).** The marker present at the suggestive QTL in Taylor 1 has a higher phenotypic means for the B allele when compared with the D allele. This difference is significant and therefore results in the presence of a QTL. The same marker in the Recent BXD RI lines has a higher phenotypic mean for the D allele, but no QTL is present within this strain set.

As an example of a single-trait analysis in the newly expanded strain set, we performed an analysis of mechanical nociception, the latency to respond to a plastic-coated smooth alligator clip placed on the tail. Previous studies of this trait in inbred mouse strains show high heritability of 0.69 (Lariviere *et al.* 2002). Analysis in MouseTrack using the RI analysis tool showed that this measure has statistically significant main effects of sex and strain (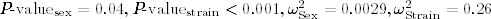 ), and interaction effects (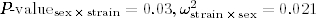 ). The variances accounted for by strain are 0.32 and 0.27 in females and males, respectively. BXD sub-population effects on females and males were non-significant (*P*-value _sub- populaton(Female)_ = 0.68*,P*-value _sub- populaton(Male)_ = 0.93). Because the main effect of sex was significant, male and female data were exported separately for analysis in GeneNetwork, which uses a single vector of strain means as input. Permutation thresholds for significant and suggestive LOD scores were 3.78 and 2.21, respectively, in males and 3.6 and 2.27, respectively, in females. Interval mapping of tail clip latency for males of the full BXD RI panel shows suggestive QTL on Chr 2 (LOD = 2.8) and 9 (LOD = 3.5), whereas suggestive QTL for females of the full panel are located on Chr 1 (LOD = 3.5), 11 (LOD = 3.0) and 17 (LOD = 2.5). Genes within the one-LOD confidence interval from QTL peaks for males (Chr 2: 77–97 Mb; Chr 9: 44–48 Mb) and females (Chr 1: 90–95 Mb and 97–107 Mb; Chr 11: 53–55 Mb; Chr 17: 78–84 Mb) were exported as positional candidate gene lists. These results, generated in the full RI panel (BXD 1–100) were compared with interval mapping results generated from previously available BXD RI lines including just the original set (Taylor I, BXD 1–32), and with exclusive use of the recent RI lines by Taylor (Taylor II, BXD 33–42) and by Peirce and colleagues (BXD 43–100). Quantitative trait locus mapping for each of the four sets (two male and two female sets) was performed. In females, suggestive QTL were detected on Chr 1 and 9 for Taylor I (Fig. 2a), and Chr 8 for the recent RI set (Fig. 2b). Mapping of tail clip latency in males showed no suggestive QTL for Taylor I (Fig. 2d) and three suggestive QTL on Chr 2, 9 and 18 for the recent RI set (Fig. 2e). The marker present at the peak of the suggestive QTL for females is gnf01.018.340 (Chr 1 at 21.43 Mb). This single nucleotide polymorphism (SNP) lies within the *Kcnq5* potassium channel gene (KCNQ5) (UCSC genome browser, http://genome.ucsc.edu/index.html, July 7, 2009). KCNQ channel (K(V)7.2-5) genes have been implicated in neuropathic pain (Gribkoff 2008). The effect at this marker across the Taylor I and recent BXD RI set indicates that while mice with the D allele have consistent phenotypic means across both sets, mice with the B allele have higher means in the Taylor I lines and lower means in the recent lines relative to the D2 allele. However, in the expanded RI set, the D2 allele is the allele with the higher effect and consequently results in a non-significant QTL (Fig. 3).

### Genetic correlation of gene expression

Genetic correlation of tail clip latency to genome-wide gene expression was performed for each of five brain tissues for which expression profiles across the BXD RI lines are in GeneNetwork, namely, whole brain, neocortex, striatum, cerebellum and hippocampus. Correlation results from each of the five brain regions were thresholded using a *P*-value of 0.001 to create coexpression candidate lists. All candidate gene lists obtained from interval mapping and genetic correlations were then uploaded into the ODE for combinatorial analysis. Maximum gene to measure (phenotype) connectivity was observed for *Slfn5* or schlafen 5 (associated with positional candidates on Chr 11 for females, male cerebellum gene expression correlates and male striatum gene expression correlates) and *Ankrd12* or ankyrin repeat domain 12 (associated with female cerebellum gene expression correlates, male cerebellum gene expression correlates and male hippocampus gene expression correlates). Other candidate genes in the interval were not found on this list of correlates, but include genes belonging to voltage-gated sodium (e.g. *Scn2b* and *Scn4b*, Tail Clip Male Positional candidates on Chr 4) or calcium channels class of genes (Bear *et al.* 2009), cholinergic receptors class of genes (e.g. Chrm4, Tail Clip Male Positional candidates on Chr 2) (Dussor *et al.* 2004) and calcitonin receptor-like genes or calcitonin gene-related peptides (e.g. *Calcrl*, Tail Clip Male Positional candidates on Chr 2) (Li *et al.* 2008), all of which have substantial literature support for involvement in nociception.

### Multi-trait QTL analysis in the expanded BXD RI lines

A total of 27 factors accounting for 73% of the total variance for the 234 variables were obtained using a maximum likelihood factor analysis of behavioral traits spanning multiple test batteries. A parallel analysis and an optimal co-ordinates analysis from the *R*/nFACTOR package both show eight informative factors. Supplementary information, Table S3, displays the factor loadings and interpretations obtained for the top 15 factors which together account for 63.5% of the total variance among the trait measures. Factors accounting for trivial amount of variances (< 1.3%) have been excluded as they would contribute little to the overall interpretation of the factors.

**Figure 4:**
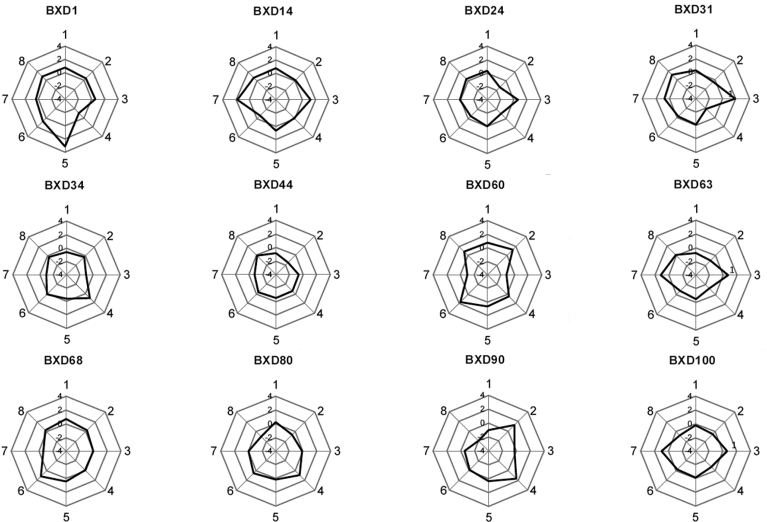
**Factor scores from individual strains plotted on a set of axes each representing a factor show a behavioral profile of each strain.** Although some strains exhibit different magnitudes of similar profiles, others are distinct. Axes labels represent the first eight factors: (1) injection stress-induced locomotor activity, (2) morphine withdrawal (3) morphine response, (4) reactivity, (5) locomotor activity in a novel environment, (6) conflict avoidance, (7) morphine activity duration and (8) cocaine sensitization. All strain profiles are available in supplementary figure.

Factor interpretations were obtained by examining high and low factor loadings of behavioral measures on factors. Significant correlations among factors exists and is expected as no factor rotation procedure was applied, as factor rotation procedures are often employed to identify distinct factors. Results indicate that measures within test batteries load onto multiple factors, thereby supporting the hypothesis that each latent factor is associated with multiple behavioral measures and each behavioral phenotype measure is determined by variation along multiple trait dimensions (Supplementary information, Table S3). Factor 1 has high factor loadings for measures almost entirely from the Cocaine test battery and can be interpreted as injection stress-induced locomotor activity. Factor 2 represents morphine withdrawal measures such as jumps, defecation and urination. Factor 3 represents morphine activity/response. Factor 4 represents reactivity measures such as startle response and anxiety-like measures. Factor 5 predominantly consists of measures pertaining to locomotor activity in a novel environment. Factor 6 contains measures pertaining to conflict avoidance. Factor 7, like Factor 3, consists of measures with high factor loadings pertaining to morphine activity, but instead of response these measures are related to duration of activity. Factor 8 consists of measures related to cocaine sensitization. Factor 9 consists of measures related to stress and anxiety such as mechanical sensitivity (von Frey threshold) and zero-maze measures and adrenal weights. Factor 10 consists of measures related to vertical activity spanning general behavior and cocaine test batteries. Factor 11 represents measures related to startle response. Factor 12 consists of measures pertaining to drug environment conditioning. Factors 13 and 14 both consist of responsivity measures, with Factor 13 describing response to sensitivity, whereas Factor 14 describes response to novelty. Lastly, Factor 15 represents anxiety related to acute stress. Factor scores from individual strains can be plotted on a set of axes representing each factor, showing a behavioral profile of each strain (Fig. 4).

Quantitative trait locus mapping of these 15 latent factors showed the presence of suggestive QTL on Chr 1, 4, 5, 6, 8 9, 10, 11, 13 and significant QTL on Chr 9 (Factor 3), Chr 10 (Factor 2) and Chr 13 (Factors 1 and 13). Results indicate that multiple factors map onto a single chromosome, for example, distal Chr 13, has suggestive QTL for Factors 1 (76–86 Mbp), 5 (78–84 Mbp, 94–98 Mbp), 6 (75–98 Mbp) and 10 (91–98 Mbp) and significant QTL for Factors 2 (78–80, 88–92 and 94–98 Mbp) and 13 (75–84 Mbp). Factor 15 also maps to Chr 13 but maps to the proximal region rather than the distal region. The Chr 13 genomic region that encompasses the above-mentioned QTL spans 23 Mbp (75–98 Mbp) with a total of 124 genes in the interval. Ninety-six of these genes are polymorphic, among the compelling candidates is *Mctp1* (multiple C2 domains, transmembrane 1). Interestingly, *Mctp1* has previously been reported to be strongly associated with bipolar disorder in individuals of European ancestry (Scott *et al.* 2009). The large number of polymorphic genes within Chr 13 QTL intervals could be one possible explanation for the fact that multiple factors map onto the same genomic region.

To determine the relationship of these factors to a substance use-related phenotype, we correlated the factor scores to phenotypic values from a previous study of ethanol self-administration (Phillips *et al.* 1994). This analysis illustrates an approach to determine which dimensions of heritable behavioral variation are most related to a specific behavioral phenotype. Correlations of alcohol preference to factor scores ranged from |*r*| = 0.076 to |*r* = 0.674, for Factor 4 for which the correlation was negative such that mice with increased startle response and anxiety-like phenotypes (manifest in our analysis as low values on the reactivity factor, Supplementary information, Table S3) had higher alcohol preference values and vice versa. This factor is regulated by a QTL on chromosome 9 which contains the *Scn4b*, an expression correlate of both ethanol withdrawal and Factor 4.

### Finding multi-dimensional extreme strains

Trait covariation implies the existence of shared genetic mediation by common polymorphisms. It is therefore expected that some strains will be extreme on a set of correlated measures, and as a group, may be useful as a starting point in selected breeding or as research models for examination of the biological substrates of extreme phenotypic deviation. The Mahalanobis distance is one such measure that can be used to detect multi-variate outliers. For example, we have detected extreme strains for composite traits spanning measures of anxiety and stress, namely, percent time spent in open quadrant (μ = 17.92 ± 8.84), percent entries in open arms (μ = 37.69 ± 12.18), percent time in center of the open field (μ = 15.75 ± 6.21) and percent time in light in light–dark box (μ = 33.64 ± 8.37). For the multi-variate set of traits analyzed, BXD 1 (high for percent time in center of open field and percent entries in open arms, intermediate for percent time spent in open quadrant, low for percent time in light in light–dark box), BXD 11 (high for percent time in light in light–dark box and percent entries in open arms, low for percent time in center of open field and percent time spent in open quadrant), BXD 16 (high for percent time in light in light–dark box and percent time in center of open field, intermediate for percent entries in open arms, low for percent time spent in open quadrant), BXD 24 (high for percent time in light in light–dark box, percent time in center of open field and percent entries in open arms, intermediate for percent time spent in open quadrant), BXD 50 (high for percent time spent in open quadrant, percent time in light in light–dark box and percent entries in open arms, intermediate for percent time in center of open field) and BXD 99 (high for percent time spent in open quadrant, percent entries in open arms and percent time in center of open field, low for percent time in light in light–dark box) were identified as significant outliers (*P*-value < 0.05). No single strain exhibited consistently high or low values across the set of anxiety-like measures, but rather had a mix of high, intermediate or low values. Therefore, each strain may possess a unique architecture of anxiety-like behavior.

### Integrating data across phenotypic batteries and comparisons to previously published studies

The multi-variate phenotypes measured in this study were obtained from multiple test batteries that were performed in multiple laboratories. Previous studies have raised the issue of robustness and stability of behavioral traits. Wahlsten *et al.* (2006) addressed this issue by comparing phenotypic data across laboratories and previously published studies for behavioral measures of locomotor activity and ethanol preference. We compared data collected for the same measure across our phenotyping laboratories and also with previously reported data for traits that were measured across multiple laboratories in the present study.

#### Saline-induced locomotor response

**Figure 5:**
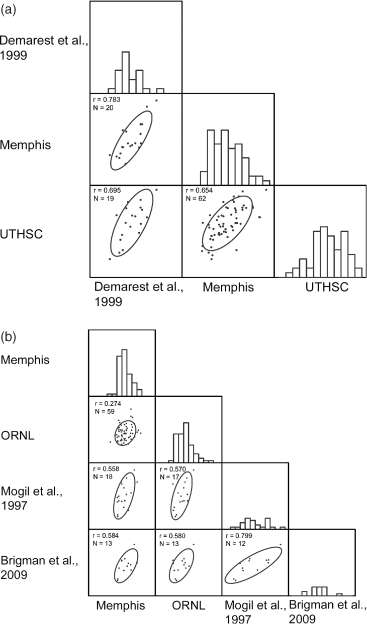
**Across trait and across studies correlations: (a) saline-induced locomotor activity: in our study two laboratories, Memphis (Cocaine) and UTHSC (Ethanol), collected data on open field locomotion following saline in 15 and 20 min on 64 BXD RI strains, respectively.** We compared these data with data collected on saline-induced locomotor response by Demarest *et al.* (1999) on 25 BXD RI strains. Results indicate that there is good correlation among data on saline-induced locomotor response across the laboratories and to a previously published study. Correlations among the *r*_Memphis,UTHSC_ = 0.556*,r*_Memphis,Demarest_ = 0.781 and *r*_Demarest,UTHSC_ = 0.695. (b) Thermal nociception: thermal nociception (hot plate latency) was preformed in two laboratories, namely, ORNL and Memphis in this study. Data collected in this study were compared with a previously published study of thermal nociception by Mogil *et al.* (1997) and Brigman *et al.* (2009). Correlations ranged from 0.274 to 0.799 (Fig. 4b). The low correlation among the data collected at the Memphis and ORNL laboratories may be attributed to the two different hot plate temperatures used.

Locomotor activity in an open field has been commonly used as a measure for exploration, novelty seeking, anxiety and predisposition to addiction to drugs of abuse. In our study, two laboratories, Memphis (Cocaine) and UTHSC (Ethanol), collected data on open field locomotion following saline in 15 and 20 min on 64 BXD RI strains, respectively. We compared these data with data collected on saline-induced locomotor response by Demarest *et al.* (1999) on 25 BXD RI strains. Results indicate that there is good correlation among data on saline-induced locomotor response across the three sets (Fig. 5a). Correlations among the *r*_Memphis,UTHSC_ = 0.556*,r*_Memphis,Demarest_ = 0.781 and *r*_Demarest,UTHSC_ = 0.695. These results confirm previous reports that locomotor activity is a highly stable trait and that saline-induced locomotor response is not only robust across laboratories but also across previously published studies.

#### Thermal nociception

Sensitivity to thermal stimuli is a complex trait that has been used as a predictor of sensitivity to analgesic drugs. Thermal nociception (hot plate latency) was performed in two laboratories, namely, ORNL and Memphis in this study. Data collected in this study were compared with a previously published study of thermal nociception by Mogil *et al.* (1997) and Brigman *et al.* (2009) (GeneNetwork RecordID: 10897). Correlations ranged from 0.274 and 0.799 (Fig. 5b). There is poor correlation among the data collected at the Memphis and ORNL laboratories and can likely be attributed to the two different hot plate temperatures used. The hot plate test was performed at 52°C at Memphis, whereas at ORNL the hot plate temperature was 54°C. Correlations increase when data are compared with previously published studies. Specifically, ORNL hot plate data have a slightly higher correlation to the Mogil study compared with the Memphis hot plate data. This is likely due to the 54°C that was employed in both the ORNL hot plate study and the Mogil study. The range of values obtained at ORNL and Memphis was lower than those of the other testing laboratories, which produced some range restriction in the observed correlation coefficients. This may be due to more consistent laboratory environmental conditions in these assays or to experimenter effects on stress-induced analgesia, both of which have been previously reported to influence thermal nociception (Chesler *et al.* 2002a,b;). These correlations are lower than the inter-laboratory correlations than those reported by Wahlsten *et al.* (2006). This may be due to the limited genetic variability in the BXD RI panel relative to a survey of the common inbred strains, but could also be due to the lower heritability of hot plate measures relative to other measures for which this type of analysis has been performed.

It should be noted that for all the above stated results, comparisons were effectively between data collected on BXD 1–32 RI strains. Given the trend of high correlations between the studies on early BXD strain sets and data collected in this study, we can expect the same for further studies that utilize the complete BXD strain set. The robustness of behavioral measures such as thermal nociception and locomotor response assayed across multiple laboratories is evident from medium to high correlations. These results reiterate the usefulness of the BXD RI strain sets as a vital reference set with respect to its reliability across laboratories and time periods.

### Combinatorial analysis of gene–phenotype associations

GeneNetwork analysis of each trait resulted in the generation of over 3500 sets of genes that are associated with trait values either via genetic correlation of gene expression in one of the five target tissues or through positional candidacy in significant or suggestive QTL. These gene–phenotype associations were integrated via a combinatorial analysis which represents the data as a bi-partite graph consisting of gene vertices and phenotype vertices. Here, a ‘phenotype’ vertex represents each list of correlates or candidates, and the gene vertices represent each list member. Edges connecting these vertices represent significant gene–phenotype associations. The union of all lists contained occurrences of approximately 33 000 genes and ESTs. However, many of these genes are represented only once, and through convergent analysis can be eliminated. By analyzing the number of phenotypes to which each gene was associated, we were able to identify those genes which most broadly and reliably related to the behavioral functions assessed. A ranked ordering of genes was generated based on each gene's connectivity to phenotypes. It should be noted that this ranking is somewhat biased by those traits which were measured in multiple assays or multiple time points. These rankings were made a part of the NeuroSNP database for supplementing addiction arrays (Saccone *et al.* 2009). The most highly connected gene was *Mef2c*; interestingly this gene also resides in the Chromosome 13 QTL interval for several behavioral factors. It should be noted that the composition of the behavioral phenotypes in this analysis will shape the results obtained. For example, some aspects of behavior such as locomotor behavior and anxiety-like behaviors have many measures in this study. As such, they are over-weighted relative to other aspects of behavior. Despite the overweighting of certain measures, the most highly connected genes are associated with approximately 150 unique traits. These traits include traits measuring anxiety, stress and pain sensitivity and span multiple test batteries namely cocaine, general behavior, morphine, ethanol and pain.

Genes that were highly connected to many of the trait measures were entered into gene set over-representation analysis using WebGestalt system which performs a hyper-geometric test for the enrichment of Gene Ontology and KEGG pathway annotations among sets of genes. Among the top most highly connected 5% of genes, significant over-representation (*P*-value < 1E–05) was observed in 30 pathways of relevance to brain and behavior. The top most highly connected 10% of genes showed significant over-representation (*P*-value < 1E–05) of 32 pathways related to brain and behavior (Table 4). These results indicate the pathways which are either candidate causes or under regulation of genetic polymorphisms that are commonly observed in the BXD population. The principle behind this approach is that genetic variation affects many genes associated with QTL candidates, and that the convergent evidence gained from multi-dimensional analyses of the same traits can highlight the most frequently associated pathways. Because these data have all been deposited in the ODE tool, users of these data can perform analyses of specific and roughly balanced subsets of the traits.

**Table 4:** List of KEGG pathways enriched among the top 5 and 10 percent of genes in the resultant gene list

	Enrichment *P*-values	
KEGG pathways	Top 5% of genes (1684)	Top 10% of genes (3368)
Adherens junction	5.42E–08	8.18E–07
Adipocytokine signaling pathway	4.43E–03	3.38E–09
Axon guidance	2.74E–11	7.72E–08
B-cell receptor signaling pathway	3.21E–06	1.47E–08
Calcium signaling pathway	1.02E–09	1.03E–11
Cell adhesion molecules (CAMs)	9.12E–09	5.02E–05
Cell cycle	1.67E–09	2.26E–04
Chronic myeloid leukemia	1.53E–05	4.53E–06
Colorectal cancer	6.16E–09	6.31E–08
Cytokine–cytokine receptor interaction	NE	1.05E–07
Focal adhesion	4.42E–09	4.96E–08
Gap junction	6.08E–08	4.57E–07
Glioma	3.24E–07	6.69E–06
GnRH signaling pathway	1.86E–11	3.67E–08
Huntington's disease	2.09E–07	8.18E–08
Insulin signaling pathway	8.06E–08	7.15E–08
Jak-signal transducer and activator of transcription (STAT) signaling pathway	7.35E–03	1.91E–06
Leukocyte trans-endothelial migration	1.38E–05	8.57E–08
Long-term depression	6.56E–08	7.13E–05
Long-term potentiation	9.80E–11	1.04E–05
MAPK signaling pathway	8.66E–15	1.43E–13
Natural killer cell-mediated cytotoxicity	1.07E–07	3.60E–14
Retroactive ligand–receptor interaction	NE	1.31E–07
Pancreatic cancer	1.97E–06	6.49E–07
Phosphatidyl-inositol signaling system	7.27E–06	1.31E–03
Regulation of actin cytoskeleton	1.13E–12	5.51E–10
T-cell receptor signaling pathway	4.48E–08	9.56E–12
Tight junction	6.36E–07	1.45E–04
Toll-like receptor signaling pathway	7.98E–05	1.73E–09
Wnt signaling pathway	1.07E–15	2.50E–09

NE, not enriched.

## Discussion

With the increased deployment of systems genetics and the availability of high-throughput molecular phenotypes in the newly expanded BXD RI strain population, there is a tremendous need for expanded complementary behavioral phenotyping in these strains. Using the high-throughput behavioral phenotyping cores established by the NIMH ENU-Neuromutagenesis Program of the TMGC, we have characterized 257 behavioral measures in the BXD RI lines. All of the primary data, strain means and candidate gene sets have been made publicly available in MouseTrack, GeneNetwork and the ODE. The availability of data that spans multiple phenotypes covering diverse aspects of behavior is intended to populate regions of sparse information within the existing BXD RI phenome, which we anticipate will be useful to future systems genetic analysis of brain and behavior.

Many of the traits in this study are mouse models of behavioral predictors of substance abuse in humans. These include stress, anxiety, novelty seeking, risk taking, impulsivity, pain sensitivity and despair. These measures were integrated with assays assessing effects of drugs such as sensitization, physiological response and withdrawal. Together these data enable a multi-dimensional genetic analysis, allowing detection of genes and genetic loci that are associated simultaneously with predisposition and drug response. This is an *en masse* approach to examining the relations among predisposing behavior and drug self-administration recently showed by Belin *et al.* (2008). Factors that predispose an individual to addiction have been grouped into three categories—environmental factors, drug-induced neural changes and genetic factors (Kreek & LaForge 2007). However, there is much interplay among them. For example, genetic variation may influence response to environmental effects in the same way as it influences drug-induced neural changes as a pleiotropic effect. Elucidating the mechanisms by which susceptibility traits relate to addiction can be achieved through continual aggregation of molecular, physiological, morphological and behavioral data in a mouse genetic reference population (Plomin *et al.* 1991).

The majority of the measures we studied are amenable for QTL mapping, as showed by high genetic effect sizes. Among these measures, 64.61% have genetic effects accounting for greater than or equal to 30% of the phenotypic variation. For those that are under weaker genetic influences, a genetic reference population offers a remedial measure to increase power by increasing the sample size within strain, especially when heritability is low. This improved phenotypic precision allows mapping of QTL for traits with heritability as low as 10% in smaller BXD populations (Belknap 1998). For lower heritability traits (*h*^2^ = 0.10), use of 58 RI strains with a within-strain sample size of 20 is equivalent to mapping in an F2 population of size 760, without the expense of genotyping individual mice and with the added value of genetic correlation across many traits. Crusio (2004) showed that maximum similarity between additive QTL effects and the correlation between a molecular marker and a strain behavioral or neuronal phenotype is achieved either when heritability approaches unity or within-strain sample sizes are infinite. At a heritability of 0.30 and a within-strain sample size of 20, QTL detection is precise and reliable with a correlation of 0.97 between a molecular marker and a behavioral phenotype. Additional power and precision can be obtained by increasing the number of strains tested as the expanded set continues toward completed inbreeding.

Sex differences and sex × strain interactions are found for a majority of the behavioral traits in this study, and as reported in previous studies (Chesler *et al.* 2002a; Valdar *et al.* 2006), sex interactions with genotype are typically more profound than main effects of sex. When significant sex differences are likely to be present, there are several approaches for considering them in further genetic analyses. Male and female phenotypic data can be analyzed separately for QTL mapping and genetic correlations. This is an approach most practically employed in tools such as GeneNetwork, which make use of a single mean value for each strain. Another approach which generates a single-trait value per strain is to regress male and female data (Fernandez *et al.* 1999). Finally, a nested analysis can be employed at each locus to appropriately consider the replication within strain and across sex within each genotype class. The latter approach reduces multiple testing concerns while retaining maximal statistical power, but requires complex permutation approaches (Peirce *et al.* 2008). By adopting a liberal bias in the detection and treatment of sex differences, the noise introduced by sex effects is reduced, allowing better detection of main effects. Further, the detection of sex-specific genetic effects shows multiple biological contexts in which to study gene effects.

Interestingly, the sex differences we observed were not just in the mean trait values of the two sexes within and across strains. Several sex differences were found in the heritability of traits, indicative of increased within strain variation relative to between strain variations. This increase in variance was not limited to females, for whom the estrous cycle is a source of variation in brain and behavior, including drug abuse (Becker & Hu 2008; Becker & Ramirez 1981; Becker *et al.* 1982). The estrous cycle is sometimes but not always a source of sex differences in behavior (Mogil *et al.* 2000; Sternberg *et al.* 2001). For some neurobehavioral phenotypes, males have a higher variability than females. This is often attributed to the social stress involved in the formation of dominance hierarchies. For example, social stressors related to fighting, such as the resident intruder paradigm have been shown to affect neurogenesis (Mitra *et al.* 2006) and cocaine-induced conditioned place preference (Mclaughlin *et al.* 2006). The sexes have also been shown to be differentially susceptible to effects of housing density and social isolation that is a part of some of these testing protocols, which may influence genetic analysis (Chesler *et al.* 2002b; Devor *et al.* 2007; Raber & Devor 2002).

Although the expanded BXD set increases the precision of the genetic map, there is concern as to whether one can simultaneously analyze the historical and new BXD RI lines, or whether systematic differences between the two sub-populations preclude this integration. In an analysis of the genetic architecture of these lines, Shifman *et al.* (2006) report 52 SNPs of 13 367 typed loci that only segregate among the new sub-populations (47 of which do not segregate in the oldest set, 5 of which do not segregate in the old lines or the first expansion). These are found on 17 chromosomes and are not tightly linked. It is especially important to note that Shifman *et al.* (2006) find these to be recent polymorphisms, not segregating among other inbred mouse strains, and therefore just an indicator of the potential recent mutation rate of 0.39% among polymorphic SNPs. It is predicted that some traits may be affected by these loci, underlying systematic differences may occur among the sub-populations. It would seem that if BXD sub-population effects are detected, the trait may be readily mapped to one of these recent polymorphisms. We have found significant sub-population effects only for the adrenal weight of males. On the basis of these results, we conclude that the new population of BXD RI lines resembles the old set, but can it be used as one integrated population?

The presence of these recent polymorphisms creates a challenge illustrated by our analysis of the tail clip phenotype. The QTL detected in the full expanded RI panel have higher LOD scores than those detected in the earlier panels. Further, these QTL appear to be more distinct from what may be ‘mirror’ loci, peaks of similar height because of non-unique strain distribution patterns when only a limited number of strains are tested, often observed when just the early set is used. Interestingly, not all loci detected in earlier panels were detected when combining these lines with the new, expanded panel. For example, the locus on chromosome 9 that was suggestive when mapped in Taylor I females had an LOD score below the suggestive threshold when mapped using the recent BXD RI set (Fig. 2a,b). Of particular interest is the locus on Chr 1, which was also suggestive in the original panel but dropped below the suggestive level in the recent BXD RI set. This could be due simply to sample size, which for RI panels is still quite low compared with other mapping populations. Therefore, mapping results are not robust to the removal of 50% of cases as was done in our analysis. However, the different results for different sub-populations more likely to illustrate the manner in which systematic differences in the new and old RI populations may conditionally affect QTL detection. Although these differences were not detected in the anova modeling as a main effect of sub-population, it is quite conceivable that these results reflect a slice through an epistatic interaction conditioned on loci that are fixed in one population or another. Our results suggest that this may even interact with sex, or that sex-specific loci may mask the detection of other QTL. Simulations can be used to determine whether the effect is due to low strain numbers or the actual composition of the population. It may prove necessary to include sub-population in the mapping model or make use of multiple-QTL modeling when using the entire combined BXD panel. Epistatic interactions with fixed polymorphisms create conditional single locus QTL effects which may vary in presence or magnitude depending on the composition of the population.

Challenges remain to the use of BXD lines including lower precision, potential of false positives due to linkage disequilibrium, finite statistical power and a limited pool of polymorphisms. These challenges are far outweighed by the ability to undertake integrative data analysis within the BXD RI lines as they serve as a common reference population. Executing similar phenotypic analysis in the Collaborative Cross (Chesler *et al.* 2008; Churchill *et al.* 2004; Iraqi *et al.* 2008; Morahan *et al.* 2008) will help address these issues and give positional refinement to the QTL identified in this study. Large cohorts of the Collaborative Cross population have been simultaneously bred, with the goal of creating well-randomized, independent RI lines.

One of the fundamental applications of these data is for the detection of QTL and identification of QTL candidate genes. Several studies have successfully achieved this goal using the BXD RI strains as a starting point, for ethanol and addiction-related phenotypes. For example, Buck and Finn (2001) identified the causative polymorphism for the alcohol withdrawal seizure originally detected in RI lines. We have employed a systematic approach to identify candidate genes by examining genes which either reside in QTL regions or are genes that are genetically correlated to the phenotype. Further reduction of positional candidates occurs through the use of complementary populations and prospective experiments. With increased availability of tools and approaches for candidate gene validation, many more QTL are being successfully identified at an increasing pace (Dipetrillo *et al.* 2005, Flint *et al.* 2005).

Human linkage analysis and genome-wide association studies are powerful tools for genetic analysis of drug and alcohol abuse (Agrawal *et al.* 2008, Uhl *et al.* 2008b). Mouse genetic analyses provide a complement to these studies by allowing access to genetic coregulation of molecular phenotypes in tissues that are not available in human clinical studies, and provide independent confirmation of regulatory loci by evaluating conservation of phenotypic association across studies (Belfer *et al.* 2006; Uhl *et al.* 2008a). Although positional candidacy is a requirement for genetic causality, our ultimate goal is to identify trait relevant genes, and therefore we include gene expression correlates. An important caveat to this approach is that the available gene expression data may come from tissues that are not necessarily the most relevant or complete substrate for the trait under consideration. These data should not be used as an exclusive filter for the candidates, but do show support for candidacy. All of the resulting genes may be candidates for human gene association studies (Saccone *et al.* 2009), because it is likely that it is the role of the gene in phenotypic variation and not the specific polymorphism that is conserved.

There have been several interesting reports of ‘behavioral hot spots' or regions of the genome that are often associated with behavior (Flint 2003). Other recent work on the structure of the mouse genome shows that there are regions of the genome which appear to have undergone selection in the tortuous history toward laboratory domestication of mice, first as pets and then as research subjects (Yang *et al.* 2007). These findings suggest that there may be a limited number of genetically variable pathways which underlie the bulk of observed neurophenotypic variation in the common mouse population. By intersecting results of genetic correlation and positional candidacy, we may identify those pathways which are repeatedly associated with neurobehavioral trait variation, and thus perhaps the pathways that underwent selection in the generation of common laboratory strains.

The development of these data and the genome-wide correlations to brain gene expression will complement gene-at-a-time approaches to addiction through the nomination of new candidate genes and the detection of multiple interacting QTL for certain traits. The phenotype-to-gene results generated from this study will aid the Knockout Mouse Project (Austin *et al.* 2004) by allowing prospective identification of the behavioral phenotypes most likely to be informative in studies of a given knock-out gene, and will therefore be useful in efforts to guide the intelligent use of costly live mouse derivation and extensive phenotyping. Further, they will become part of the foundation for deeper systems genetic analyses of drug abuse in the BXD RI lines.

## Supporting Information

**Figures S1–S3:** Factor score plots for the full BXD RI panel.

**Table S1:** Summary of Strain × Sex effects for all measures.

**Table S2:** Accession IDs for Gene Network and Ontological Discovery Environment records.

**Table S3:** Factor loadings and interpretations.

As a service to our authors and readers, this journal provides supporting information supplied by the authors. Such materials are peer-reviewed and may be re-organized for online delivery, but are not copy-edited or typeset. Technical support issues arising from supporting information (other than missing files) should be addressed to the authors.
