# Supplementary figures and images for: High-throughput behavioral phenotyping in the expanded panel of BXD recombinant inbred strains
Source: Genes Brain Behav. 2010 Mar;9(2):129–59. doi: 10.1111/j.1601-183X.2009.00540.x (PMC2855868; doi:10.1111/j.1601-183X.2009.00540.x)

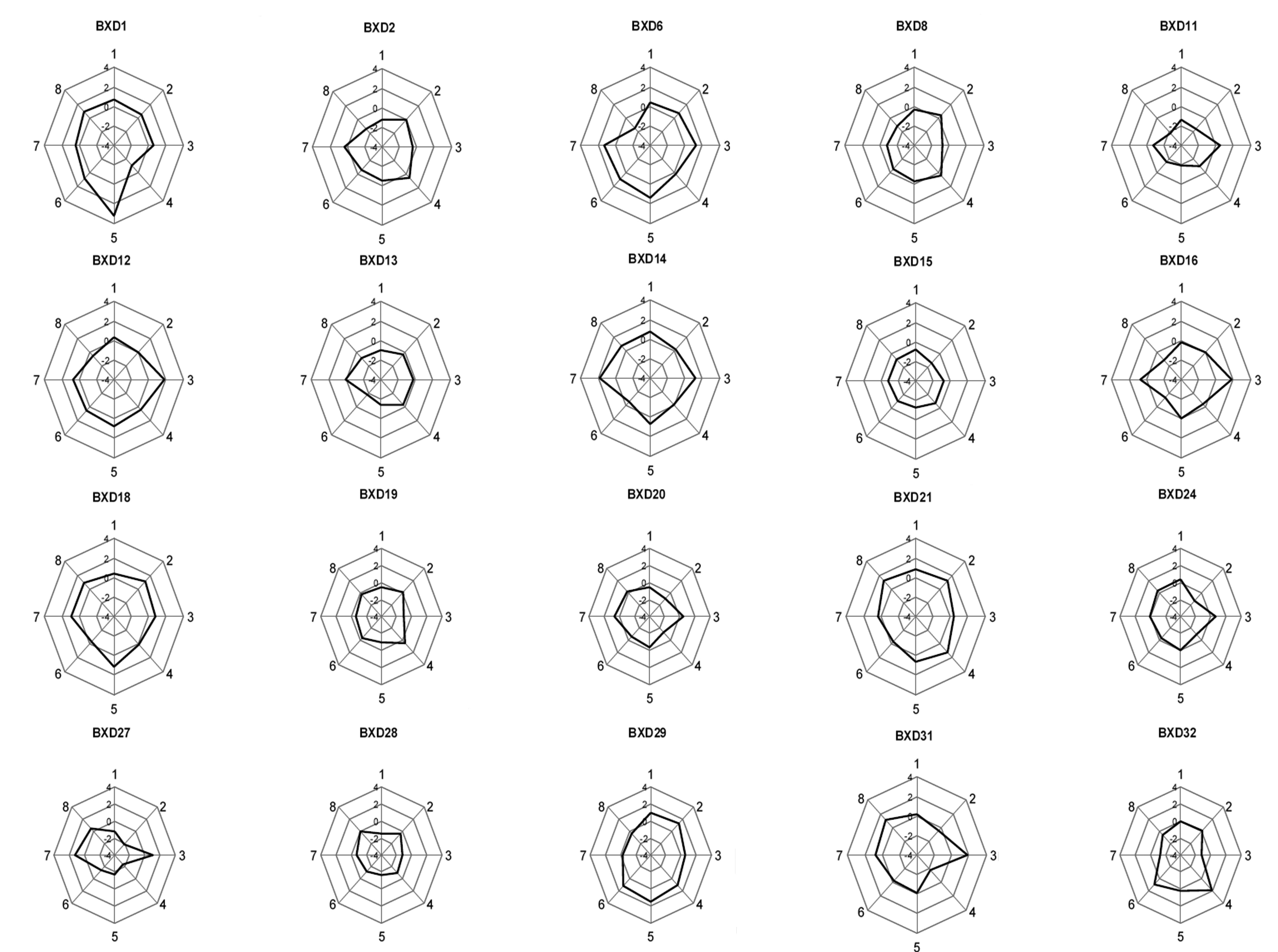

Supplement: Supplementary file 1 [file gbb0009-0129-SD1.jpg]

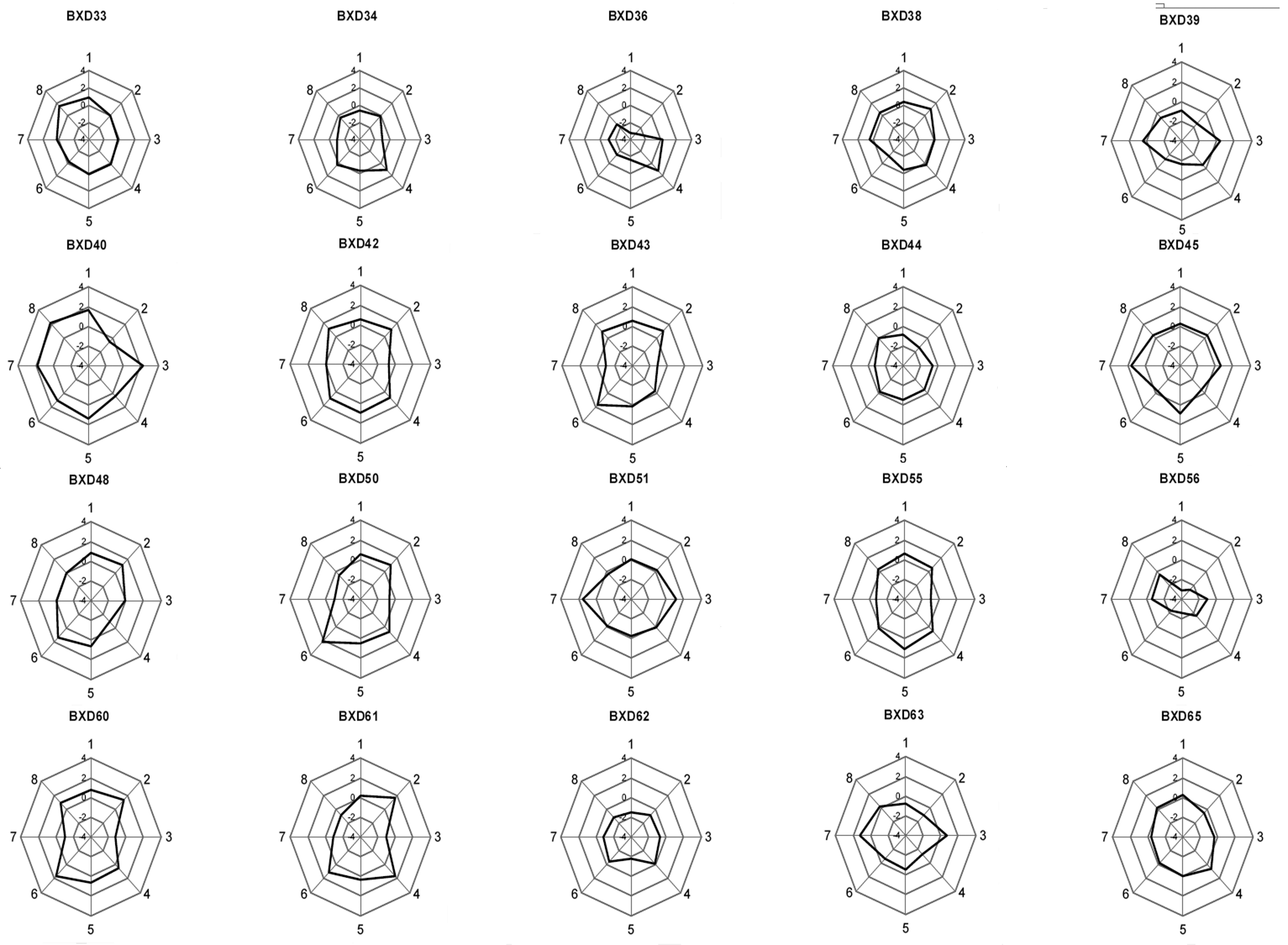

Supplement: Supplementary file 2 [file gbb0009-0129-SD2.jpg]

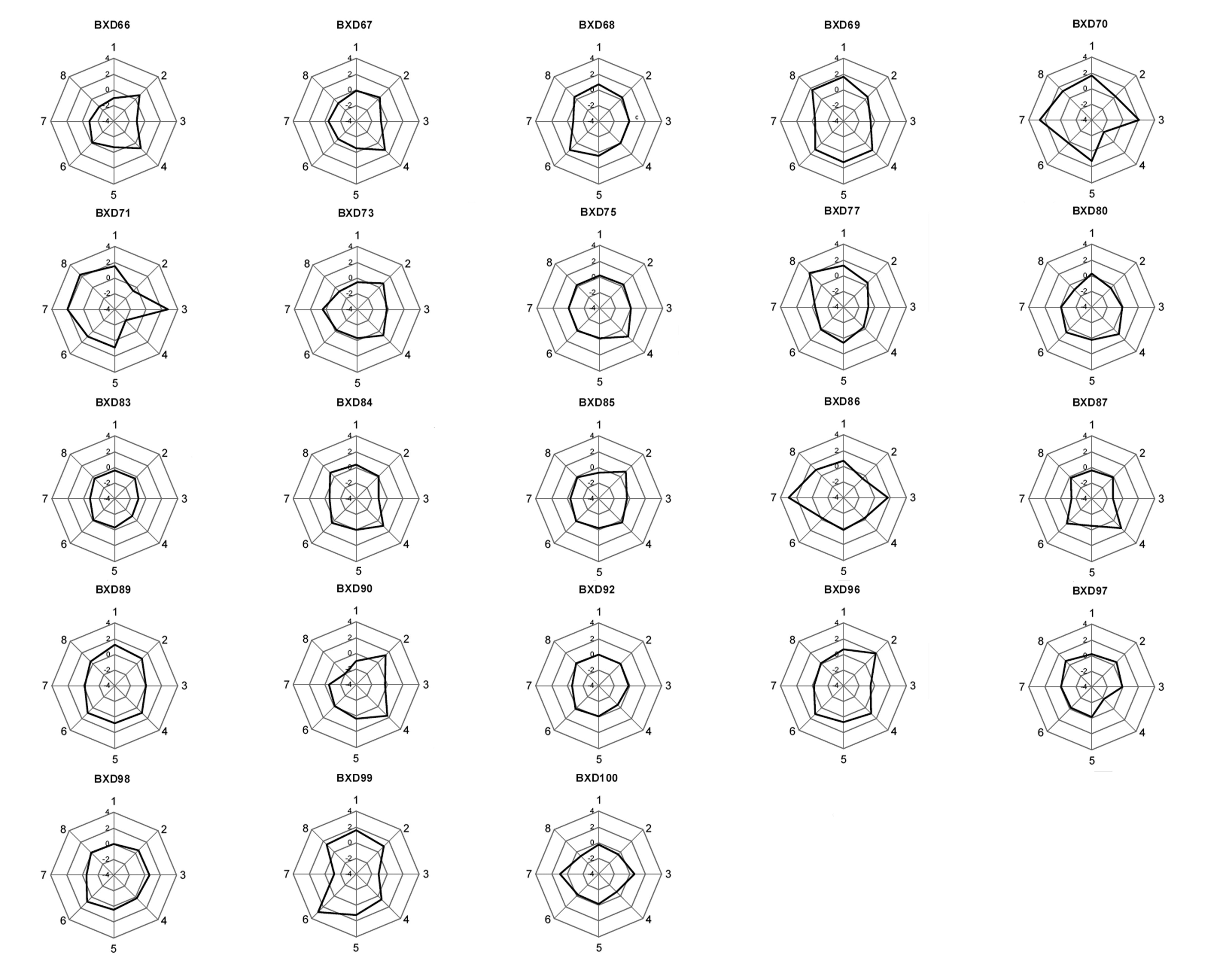

Supplement: Supplementary file 3 [file gbb0009-0129-SD3.jpg]
